# Signaling metabolite succinylacetone activates HIF-1**α** and promotes angiogenesis in *GSTZ1*-deficient hepatocellular carcinoma

**DOI:** 10.1172/jci.insight.164968

**Published:** 2023-10-31

**Authors:** Huating Luo, Qiujie Wang, Fan Yang, Rui Liu, Qingzhu Gao, Bin Cheng, Xue Lin, Luyi Huang, Chang Chen, Jin Xiang, Kai Wang, Bo Qin, Ni Tang

**Affiliations:** 1 Key Laboratory of Molecular Biology for Infectious Diseases (Ministry of Education), Institute for Viral Hepatitis, Department of Infectious Diseases, The Second Affiliated Hospital;; 2 Department of Geriatrics, The First Affiliated Hospital;; 3 Department of Infectious Diseases, The First Affiliated Hospital;; 4 Department of Gastrointestinal Surgery, The Second Affiliated Hospital; and; 5 Institute of Life Sciences, Chongqing Medical University, Chongqing, China.

**Keywords:** Oncology, Liver cancer

## Abstract

Aberrant angiogenesis in hepatocellular carcinoma (HCC) is associated with tumor growth, progression, and local or distant metastasis. Hypoxia-inducible factor 1α (HIF-1α) is a transcription factor that plays a major role in regulating angiogenesis during adaptation of tumor cells to nutrient-deprived microenvironments. Genetic defects in Krebs cycle enzymes, such as succinate dehydrogenase and fumarate hydratase, result in elevation of oncometabolites succinate and fumarate, thereby increasing HIF-1α stability and activating the HIF-1α signaling pathway. However, whether other metabolites regulate HIF-1α stability remains unclear. Here, we reported that deficiency of the enzyme in phenylalanine/tyrosine catabolism, glutathione *S*-transferase zeta 1 (*GSTZ1*), led to accumulation of succinylacetone, which was structurally similar to α-ketoglutarate. Succinylacetone competed with α-ketoglutarate for prolyl hydroxylase domain 2 (PHD2) binding and inhibited PHD2 activity, preventing hydroxylation of HIF-1α, thus resulting in its stabilization and consequent expression of vascular endothelial growth factor (VEGF). Our findings suggest that GSTZ1 may serve as an important tumor suppressor owing to its ability to inhibit the HIF-1α/VEGFA axis in HCC. Moreover, we explored the therapeutic potential of HIF-1α inhibitor combined with anti–programmed cell death ligand 1 therapy to effectively prevent HCC angiogenesis and tumorigenesis in *Gstz1*-knockout mice, suggesting a potentially actionable strategy for HCC treatment.

## Graphical abstract

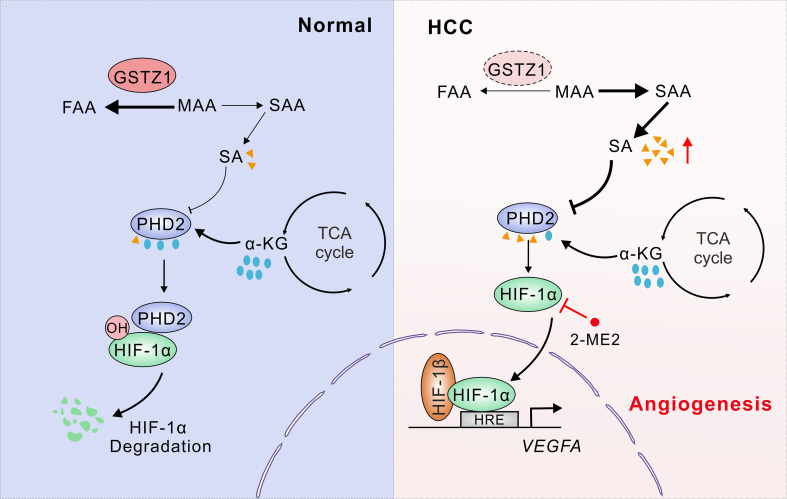

## Introduction

Angiogenesis is the physiological process in which new blood vessels are formed from preexisting blood vessels. Vascular endothelial growth factor (VEGF) is one important angiogenic factor that is involved in the regulation of angiogenesis in hepatocellular carcinoma (HCC) (1). Current treatment options for advanced, unresectable HCC with malignant angiogenesis are restricted to tyrosine kinase inhibitors and immune checkpoint inhibitors, with limited efficacy in the majority of patients with HCC (2). Therefore, the identification of molecular biomarkers to improve clinical decision-making and of novel antiangiogenesis strategies for HCC therapy remains a high priority.

Hypoxia, in cooperation with oncogenes, reprograms metabolic pathways in tumor cells to support their proliferation and survival. Hypoxia-inducible factor 1α (HIF-1α) is the main transcription factor involved in the adaptation to hypoxic environments in cancer cells, contributing to the regulation of metabolism, angiogenesis, cell survival, and drug resistance (3), thus making it an appealing target for tumor therapy. As a central node to coordinate different metabolic processes, HIF-1α mediates adaptive metabolic responses of cells to hypoxia by increasing energy flux through glycolysis and decreasing the entry of glycolytic carbon into the tricarboxylic acid (TCA) cycle (4). HIF-1α target genes, including *GLUT1*, *HK2*, *PDK1*, *PFKFB*, and *VEGFA*, are pivotal in regulating tumor metabolism, metastasis, and angiogenesis (5–8). The stability of HIF-1α is mainly regulated by ubiquitination and deubiquitination (9). Under normoxic conditions, HIF-1α is hydroxylated at conserved proline residues by HIF prolyl hydroxylase domain (PHD) proteins, depending on the availability of its cofactors, O_2_, Fe^2+^, ascorbate, and α-ketoglutarate (α-KG). HIF-1α hydroxylation induces its binding to the von Hippel-Lindau protein, ubiquitination, and subsequent proteasomal degradation (10, 11). Under hypoxic conditions the PHD enzymes become inactive, and instead of being degraded HIF-1α forms a heterodimer with the HIF-1β subtype, which activates the transcription of downstream genes (12). Many studies provide evidence that metabolites can regulate HIF-1α stability (13, 14), but the exact mechanisms by which HIF-1α levels are coordinated by a complex interplay between oxygen and metabolic signals are not fully understood.

Metabolic reprogramming is a major hallmark of tumor progression. Tumor cells undergo huge metabolic changes in order to survive in a nutrient- and oxygen-deprived tumor microenvironment (TME) and meet the needs of rapidly dividing cells for energy, biosynthesis, and redox homeostasis (15, 16). Genetic defects in TCA cycle enzymes, such as succinate dehydrogenase and fumarate hydratase, result in a blockade of the TCA cycle and an abnormal accumulation of succinate and fumarate. Both are structural analogs of α-KG and inhibit PHD2 activity, which in turn stabilizes HIF-1α (14, 17). Due to the limited understanding of the crosstalk among various metabolic and carcinogenic pathways, whether other oncometabolites regulate HIF-1α expression and function in HCC remains elusive.

Mutations in phenylalanine/tyrosine (Phe/Tyr) catabolism enzymes cause different metabolic diseases (18, 19). Patients with hereditary tyrosinemia type 1, the most serious disease among the Phe/Tyr metabolic disorders are at a high risk of HCC (20). Glutathione *S*-transferase zeta 1 (*GSTZ1*) is the penultimate enzyme of Phe/Tyr catabolism, which occurs mainly in the liver (21). Our group has reported that loss of *GSTZ1* leads to succinylacetone (SA) accumulation and poor clinical outcomes in HCC (22). As a new carcinogenic metabolite, SA is structurally similar to α-KG. Thus, we speculated that dysregulated metabolites due to *GSTZ1* deficiency may be associated with the hypoxic TME driving hepatic tumorigenesis. In this study, we found that *GSTZ1* deficiency promoted HCC angiogenesis both in vivo and in vitro. HIF-1α inhibition combined with anti–programmed cell death ligand 1 (anti–PD-L1) therapy effectively prevented HCC growth in *Gstz1*-knockout mice, providing an alternative strategy for HCC treatment.

## Results

### GSTZ1 expression is negatively correlated with VEGFA in hepatoma cell lines and human HCC tissues.

**Figure 1:**
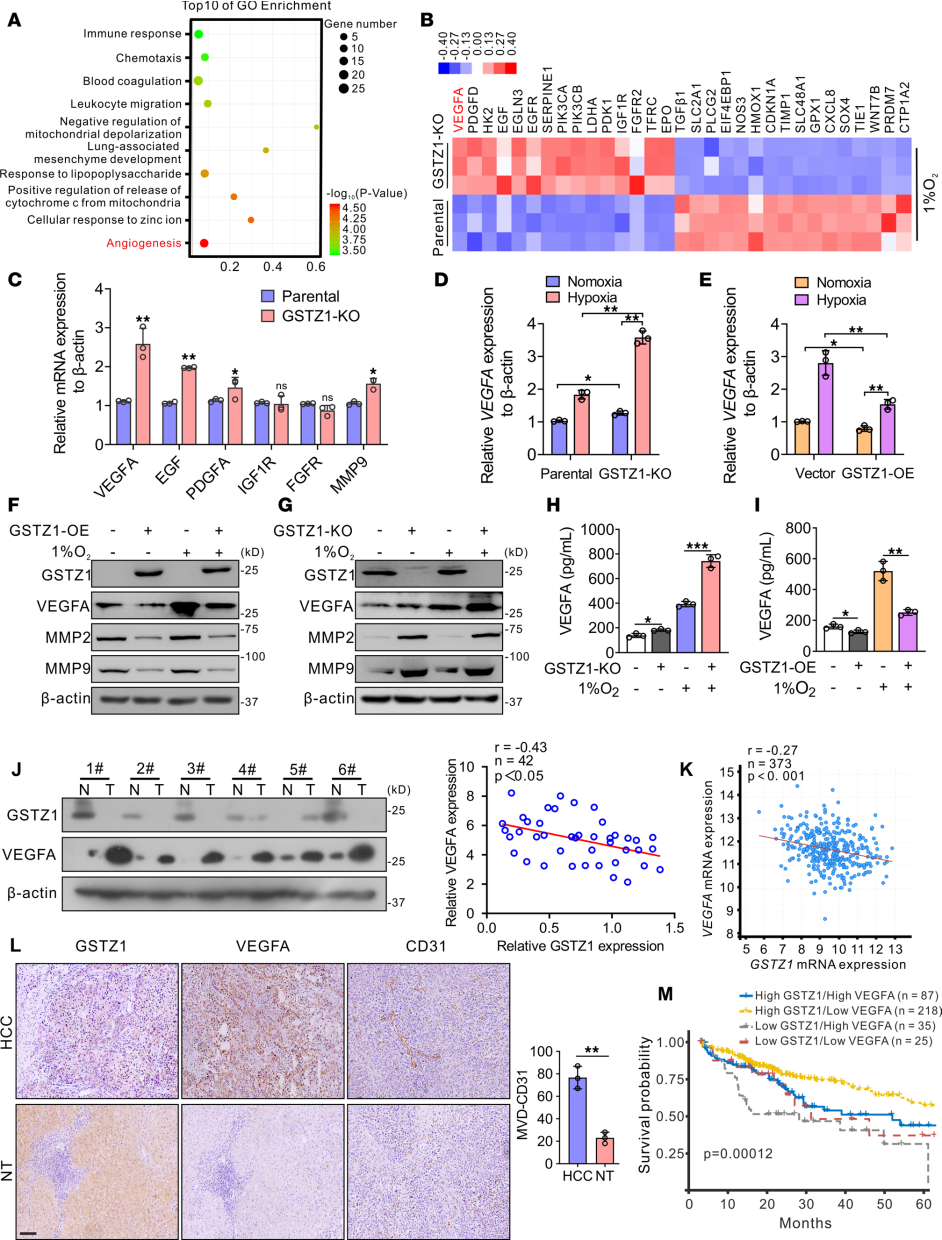
GSTZ1 expression is negatively correlated with VEGFA in HCC cell lines and tissue. (**A**) Gene set enrichment analysis is used to categorize the pathways that are significantly altered in *GSTZ1*-KO under hypoxia conditions, with angiogenesis signaling highlighted. (**B**) RNA-Seq results reveal that *GSTZ1*-KO promotes the angiogenesis pathway in HepG2 cells under hypoxia conditions. (**C**) Differential expression of angiogenesis-related genes. Data represent mean ± SEM for 3 independent experiments. (**D** and **E**) Relative expression of *VEGFA* mRNA level under indicated treatments. Data represent mean ± SEM for 3 independent experiments. (**F** and **G**) Western blotting shows the VEGFA, MMP2, and MMP9 expression levels under indicated treatments. (**H** and **I**) ELISA measurements of the VEGFA protein level in the culture medium of *GSTZ1*-KO HepG2 cells and *GSTZ1*-OE Huh7 cells under hypoxia or normoxia for 12 hours (*n* = 3 in each group). (**J**) GSTZ1 and VEGFA protein expression in 42 HCC and paired nontumor tissue specimens. (**K**) Correlation analysis of *GSTZ1* and *VEGFA* mRNA expression is conducted using data from 373 patients with HCC included in TCGA LIHC data set. (**L**) Representative GSTZ1, VEGFA, and CD31 IHC staining in 3 cases of HCC and paired nontumor tissues (scale bar = 200 μm). (**M**) Overall survival in HCC patients with high (> 25 percentile) or low (≤ 25 percentile) mRNA expression of *GSTZ1* and *VEGFA*, based on TCGA data. Data are shown as the mean ± SEM. Statistical analysis was performed using 2-tailed unpaired Student’s *t* test (**C**, **H**, **I**, and **L**), 1-way ANOVA with Tukey’s test (**D** and **E**), Pearson *r* test (**J** and **K**) or Gehan-Breslow-Wilcoxon test (**M**); *P < 0.05, **P < 0.01, ***P < 0.001. N, normoxia; H, hypoxia; HCC, hepatocellular carcinoma; KO, knockout; N, nontumor; T, tumor; TCGA LIHC, The Cancer Genome Atlas Liver Hepatocellular Carcinoma.

Our previous studies found that GSTZ1 was significantly downregulated in HCC (22, 23). To investigate the mechanisms underlying *GSTZ1* deficiency in HCC, the effect of HBV infection on GSTZ1 expression was explored. Interestingly, we found that HBV1.1 or the main pathogenic X (HBx) protein markedly reduced the mRNA and protein expression of GSTZ1, while HBx-deficient HBV or other HBV components did not have a significant effect on GSTZ1 expression. The above data indicated that HBV infection, especially the HBx protein, is closely related to GSTZ1 downregulation in the liver (Supplemental Figure 1, A and B; supplemental material available online with this article; https://doi.org/10.1172/jci.insight.164968DS1). To characterize *GSTZ1*-dependent global changes in the transcriptome under hypoxia, we conducted a genome-wide RNA-sequencing (RNA-Seq) analysis. First, we established *GSTZ1*-knockout (*GSTZ1*-KO) HepG2 cell lines using the CRISPR/Cas9 system. Total RNA was isolated from *GSTZ1*-KO and parental HepG2 cells (control) maintained under 1% O_2_ concentration for 12 hours and subjected to sequencing. Based on the significance criterion (*P* value), a total of 2,605 transcripts were significantly altered in *GSTZ1*-KO cells as compared with parental cells (fold-change > 1.5 or < 0.667, FDR < 0.05, *n* = 590; Supplemental Figure 1C). Gene Ontology analysis revealed that angiogenesis, immune response, and toxin metabolic processes were activated in *GSTZ1*-KO cells under a hypoxic condition (Figure 1A). The heatmap showed that the differentially expressed genes were enriched in the angiogenesis pathways. Notably, the expression of *VEGFA*, a key player in HCC angiogenesis (24), was significantly upregulated in response to *GSTZ1* deficiency (Figure 1B). Furthermore, quantitative reverse transcription PCR (qRT-PCR) data revealed that *GSTZ1* deficiency promoted the expression of *VEGFA* and *MMP9* in *GSTZ1*-KO HepG2 cells under hypoxia (Figure 1C). Moreover, both mRNA and protein expression of VEGFA were upregulated in *GSTZ1*-KO HepG2 cells, while they were downregulated in *GSTZ1*-overexpressing (*GSTZ1*-OE) Huh7 cells both under normoxia and hypoxia; these changes were more pronounced under hypoxia (Figure 1, D–I). *GSTZ1* silencing promoted MMP2 and MMP9 protein expression and vice versa (Figure 1, F and G).

Next, we assessed GSTZ1 and VEGFA protein levels in 42 paired HCC and adjacent normal tissues (Figure 1J and Supplemental Figure 1D). GSTZ1 protein expression levels were significantly lower in HCC tissues than in adjacent normal tissues, whereas VEGFA protein expression levels were notably higher in HCC tissues than in adjacent normal tissues; furthermore, GSTZ1 expression was significantly negatively correlated with VEGFA expression (*r* = –0.43, *P* < 0.05) (Figure 1J). In addition, analysis of TCGA database revealed a negative correlation between the mRNA levels of *GSTZ1* and *VEGFA* in 373 patients with HCC (*r* = –0.27, *P* < 0.001) (Figure 1K). We also detected the protein expression of GSTZ1, VEGFA, and CD31 (indicator of microvessel density, MVD) using immunohistochemistry (IHC) staining and found a significant negative correlation between GSTZ1 and VEGFA expression and between GSTZ1 and CD31 expression (Figure 1L and Supplemental Figure 1E). Kaplan-Meier survival curve analysis showed that patients with low expression of *GSTZ1* and high expression of *VEGFA* had the lowest overall survival (*P* = 0.00012, Figure 1M). Together, these data illustrate that GSTZ1 may play a negative role in angiogenesis in HCC cells and tissues.

### GSTZ1 suppresses HCC angiogenesis in vitro and in vivo.

**Figure 2:**
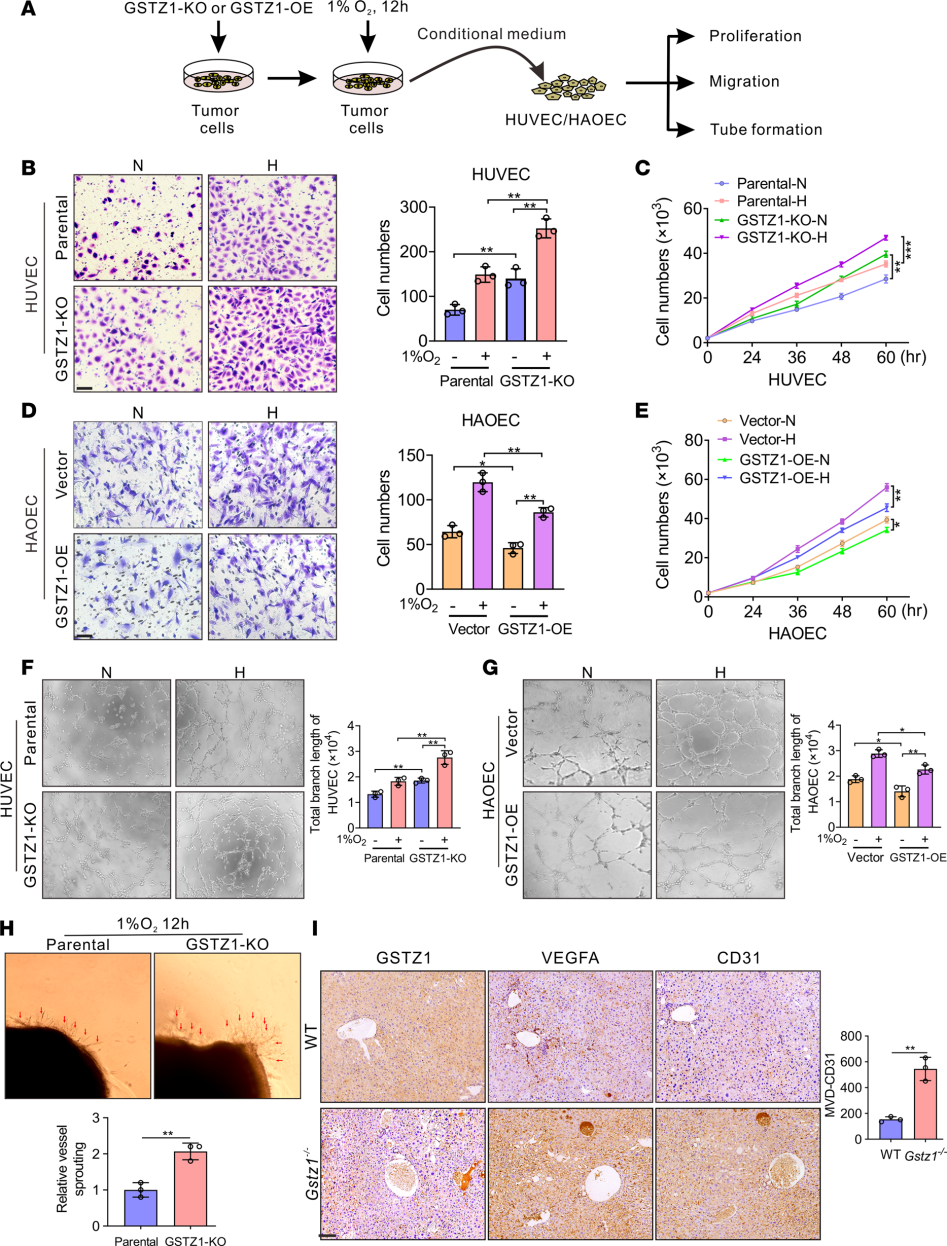
GSTZ1 suppresses HCC angiogenesis in vitro and in vivo. (**A**) Schematic illustration of the sample treatment and collection. The *GSTZ1*-KO HepG2 cells and *GSTZ1*-OE Huh7 cells are exposed to either 21% O_2_ or 1% O_2_ for 12 hours. The conditioned medium (CM) is harvested for culturing the primary HUVECs and HAOECs. (**B** and **D**) Migration by Transwell assays (scale bar = 10 μm). (**C** and **E**) Cell growth curves. (**F** and **G**) Tube formation assays in HUVECs in the culture medium of parental and *GSTZ1*-KO HepG2 cells cultured under hypoxia or normoxia for 12 hours. (**H**) Aortic ring sprouting assay. Aortic segments are harvested from C57BL/6 mice. Aortic segments in Matrigel are treated for 8 days with the culture supernatant of CM. Arrows point at the new sprouts. The original magnification of **F** and **G** is 10×, and the original magnification of **H** is 4×. (**I**) The representative IHC staining images (scale bar = 200 μm) of GSTZ1 and VEGFA and the number of microvessels of WT and *Gstz1^–/–^* mice. Microvessels are measured using the CD31 IHC staining. Data are shown as mean ± SEM (*n* = 3 in each group). Statistical analysis was performed using 1-way ANOVA with Tukey’s test (**B**, **D**, **F**, and **G**), 2-way ANOVA with Bonferroni’s test (**C** and **E**) or 2-tailed unpaired Student’s *t* test (**H** and **I**); *P < 0.05, **P < 0.01, ***P < 0.001.

In light of the above findings, we subsequently explored the biological functions of *GSTZ1*-reprogrammed endothelial cells in tumor progression in the TME. Conditional media from *GSTZ1*-KO HepG2 cells and *GSTZ1*-OE Huh7 cells after exposure to normoxia (21% O_2_ for 12 hours) and hypoxia (1% O_2_ for 12 hours) were collected and incubated with human umbilical vein endothelial cells (HUVECs) and human aortic ECs (HAOECs) to perform the Transwell, proliferation, wound-healing, and tube formation assays in vitro (Figure 2A). Conditioned medium (CM) harvested from *GSTZ1*-KO HepG2 cells promoted proliferation, migration, and tube formation in primary HUVECs and HAOECs compared with CM collected from parental cells. Conversely, CM harvested from *GSTZ1*-OE cells inhibited HUVEC and HAOEC proliferation, migration, and angiogenesis (Figure 2, B–G, and Supplemental Figure 2, A–G). Furthermore, we validated the sprouting of vessels from C57BL/6 mouse aortic rings and found that CM from *GSTZ1*-KO HepG2 cells promoted budding of mouse aortic rings compared with the control (Figure 2H). We further investigated the relationship between GSTZ1 and HCC angiogenesis in vivo. The diethylnitrosamine (DEN)/CCl_4_-induced mouse model of liver cancer was established as previously described (22). We found that *Gstz1^–/–^* mouse tumors displayed higher VEGFA and CD31 expression than the wild-type (WT) group. Moreover, the MVD of *Gstz1^–/–^* mouse tumors was significantly increased (Figure 2I and Supplemental Figure 2H). Taken together, these results verify that GSTZ1 suppresses HCC angiogenesis in vitro and in vivo.

### GSTZ1 suppresses HCC angiogenesis via inactivating the HIF-1α signaling pathway.

**Figure 3:**
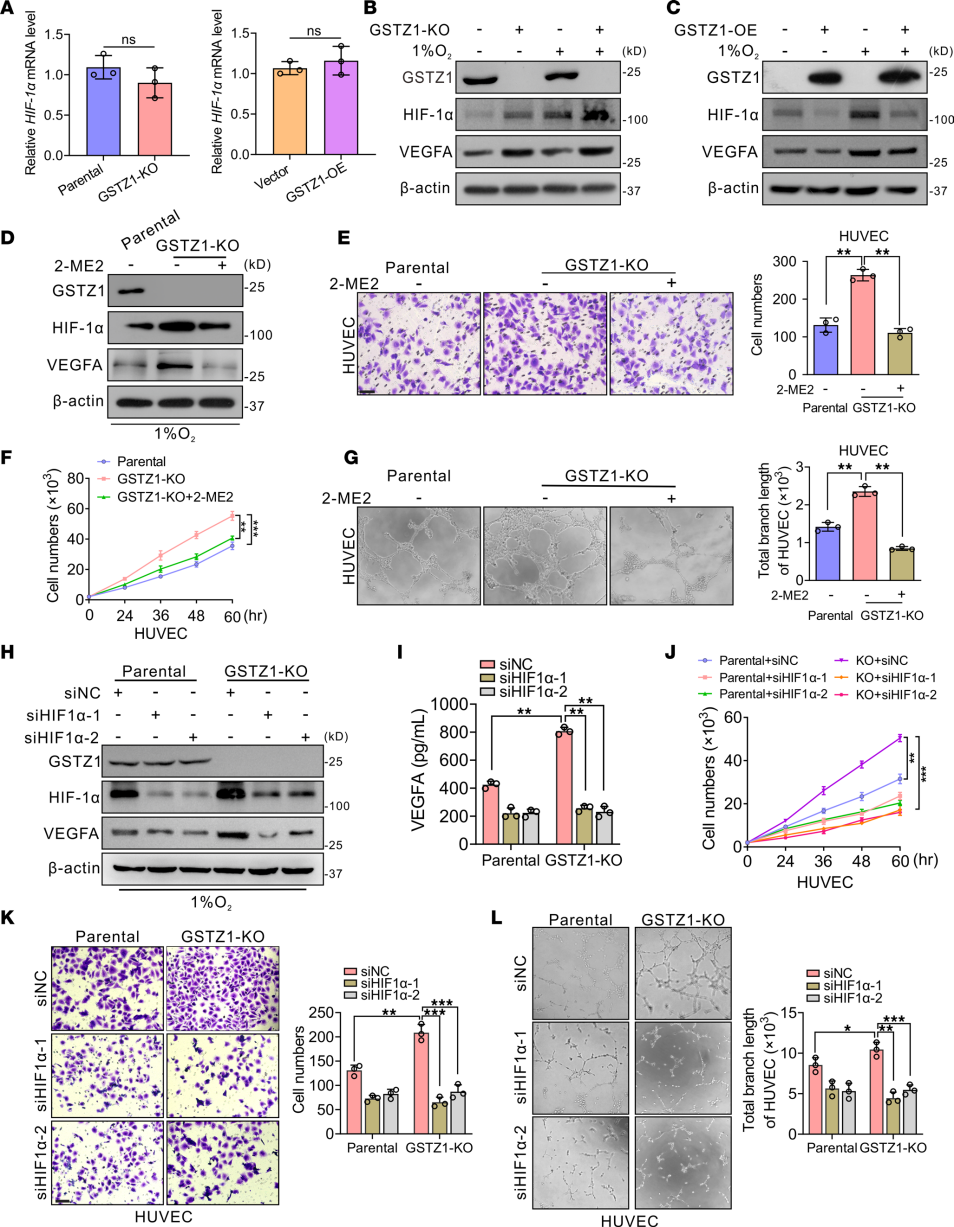
GSTZ1 suppresses HCC angiogenesis by inactivating the HIF-1α signaling pathway. (**A**) *HIF-1*α mRNA level under hypoxia for 12 hours in *GSTZ1*-KO HepG2 cells and in *GSTZ1*-OE Huh7 cells. (**B** and **C**) Western blot analysis shows the expression of GSTZ1, HIF-1α, and VEGFA proteins in HepG2 with *GSTZ1*-KO cells and Huh7 with *GSTZ1*-OE cells, which were incubated under normoxia or hypoxia for 12 hours. (**D**) The protein expression level of GSTZ1, HIF-1α, and VEGFA proteins in HepG2 *GSTZ1*-KO cells treated with HIF-1α inhibitor 2-Methoxyestradiol (2-ME2) under hypoxia for 12 hours. (**E**) Migration by Transwell assays (scale bar = 10 μm). (**F**) Cell growth curves. (**G**) Tube formation assays in HUVECs. (**H**) Western blot shows protein expression of HIF-1α, VEGFA, and GSTZ1 in HepG2 with *GSTZ1*-KO cells ectopically expressing siNC or siHIF-1α under hypoxia for 12 hours. (**I**) VEGFA protein levels in the culture medium of HepG2 with *GSTZ1*-KO cells and parental cells ectopically expressing siNC or siHIF-1α under hypoxia conditions for 12 hours. (**J**) Cell growth curves under hypoxia. (**K**) Migration by Transwell assays (scale bar = 10 μm). (**L**) Tube formation assays in HUVECs with the culture medium of HepG2 with *GSTZ1*-KO cells and parental cells ectopically expressing siNC or siHIF-1α under hypoxia for 12 hours, respectively. For Western blotting, 30–50 μg of protein is loaded per well. The original magnification of **G** and **L** is 10×. Values represent the mean ± SEM (*n* = 3 in each group). The qRT-PCR data are determined from 3 independent experiments. Statistical analysis was performed using 2-tailed unpaired Student’s *t* test (**A**), 1-way ANOVA with Tukey’s test (**E**, **G**, **I**, **K**, and **L**) or 2-way ANOVA with Bonferroni’s test (**F** and **J**); *P < 0.05, **P < 0.01, ***P < 0.001. siNC, negative control.

HIF-1α is a regulated subunit of the transcription factor HIF-1 that forms a heterodimer with HIF-1β and recognizes hypoxia-responsive elements. VEGFA functions as a hypoxia-inducible angiogenic factor (25). GSTZ1 could decrease the protein but not mRNA level of HIF-1α under hypoxia in hepatoma cells (Figure 3, A–C), indicating that GSTZ1 may negatively modulate HIF-1α expression at the protein level. To further explore whether HIF-1α is responsible for the increased VEGFA expression associated with *GSTZ1* deficiency, the HIF-1α inhibitor 2-Methoxyestradiol (2-ME2), which exhibits anti–HIF-1α activity by preventing its nuclear accumulation (26), as well as siRNAs or single-guide RNAs targeting HIF-1α, were used. Pharmacological inhibiting of HIF-1α suppressed the production of VEGFA in *GSTZ1*-KO HepG2 cells (Figure 3D). *GSTZ1*-KO promoted HUVECs’ proliferation, migration, and angiogenesis capacity, while angiogenesis caused by *GSTZ1* deficiency was inhibited by 2-ME2, indicating the essential role of HIF-1α in HCC progression upon *GSTZ1* depletion (Figure 3, E–G, and Supplemental Figure 3, A–C). Meanwhile, HIF-1α silencing or depletion almost completely diminished the increase in VEGFA production induced by *GSTZ1* deficiency (Figure 3, H and I, and Supplemental Figure 3, D and E). Functional studies further suggested that HIF-1α silencing or depletion reduced HUVEC and HAOEC proliferation, migration, and angiogenesis capacity caused by *GSTZ1* deficiency, concomitant with the decreased VEGFA expression (Figure 3, J–L, and Supplemental Figure 4, A–F). These data suggest that tumor-associated angiogenesis induced by *GSTZ1* depletion is related to the HIF-1α/VEGFA pathway. Collectively, these results support the hypothesis that *GSTZ1* deficiency promotes HCC progression and angiogenesis by activating the HIF-1α signaling pathway to induce VEGFA expression.

### Targeting HIF-1α alleviates the tumor-promoting effect of GSTZ1 deficiency in orthotopic mouse models of HCC.

**Figure 4:**
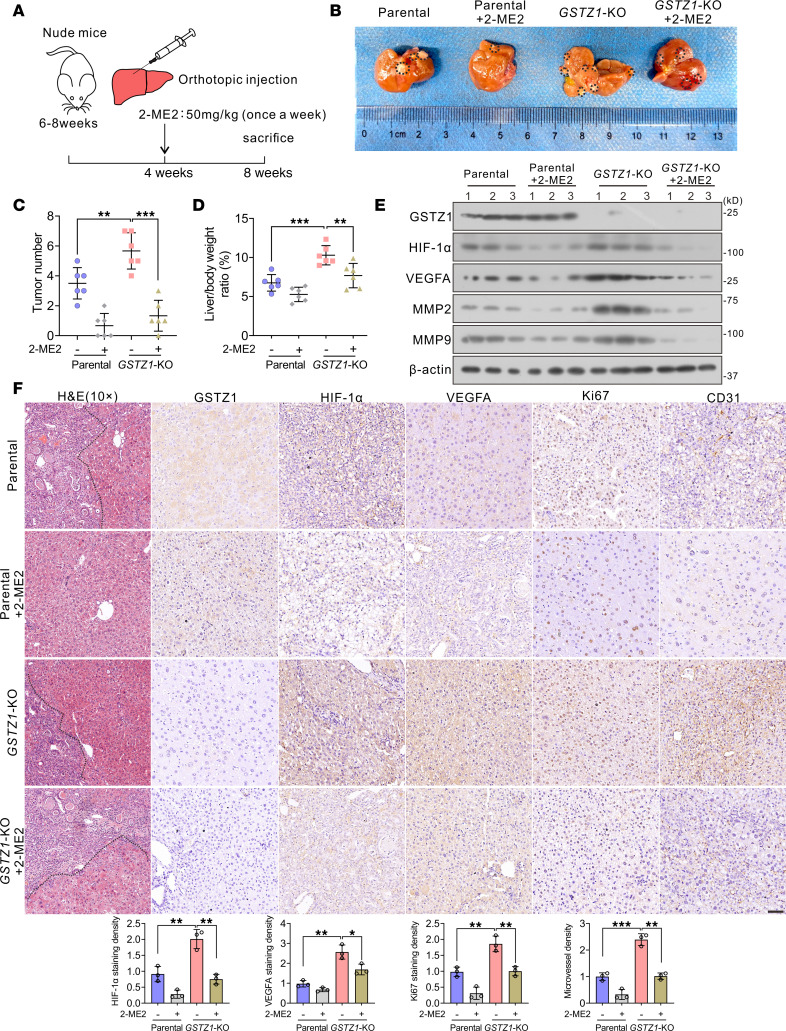
Targeting HIF-1α alleviates the tumor-promoting effect of GSTZ1 deficiency in orthotopic mouse models of HCC. (**A**) Schematic representation of the experimental design for procedures involving nude mouse orthotopic injection. (**B**) Gross appearance of liver tumors. The black circle represents nodules of the tumors. (**C** and **D**) Tumor numbers (**C**) and tumor liver/body weight ratio (**D**). Data are shown as mean ± SEM (*n* = 6 mice per group). (**E**) Western blots show protein expression of GSTZ1, HIF-1α, VEGFA, MMP2, and MMP9 in 3 groups of liver tumors. (**F**) The representative IHC staining images (scale bar = 50 μm) and the expression staining density of HIF-1α, VEGFA, MVD, and Ki67 in the tumor tissues of orthotopic hepatocarcinoma xenografts. The immunostaining signal intensity is quantitatively analyzed using ImageJ software (NIH), and data are presented as the means ± SEM from 3 independent experiments. Statistical analysis was performed using 1-way ANOVA with Tukey’s test; *P < 0.05, **P < 0.01, ***P < 0.001.

Considering the important role of HIF-1α in angiogenesis, we next investigated the effect of GSTZ1 on HIF-1α expression, tumor progression, and metastasis in orthotopic mouse models of HCC (Figure 4A). The results showed that *Gstz1*-KO promoted HCC progression, whereas 2-ME2 treatment significantly decreased the proliferation of parental and *Gstz1*-KO cells in vivo based on tumor number and liver-to-body weight ratio (Figure 4, B–D). The protein levels of HIF-1α, VEGFA, MMP2, and MMP9 were increased in *Gstz1*-KO mice. However, the HIF-1α inhibitor 2-ME2 abrogated the increase in VEGFA, MMP2, and MMP9 induced by *GSTZ1* deficiency in orthotopic mouse models, revealing that inhibition of HIF-1α with 2-ME2 substantially reversed tumor growth, MVD, and progression in *Gstz1*-KO mice (Figure 4, E and F). These results revealed that targeting HIF-1α prevented the tumor-promoting effect of *GSTZ1* deficiency.

### Loss of GSTZ1 results in SA accumulation and HIF-1α activation.

**Figure 5:**
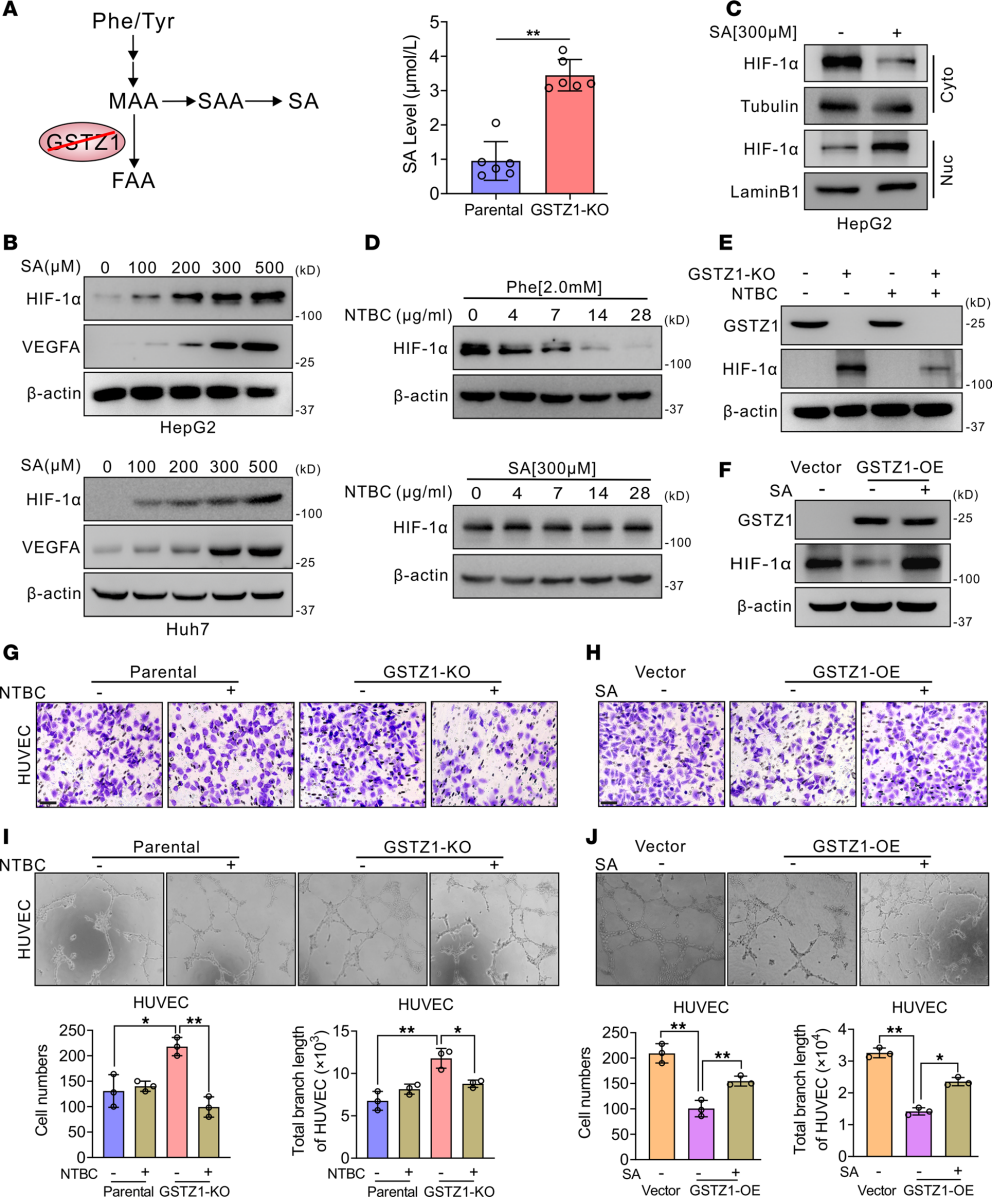
Loss of GSTZ1 results in SA accumulation and HIF-1α activation. (**A**) Schematic representation of the phenylalanine and tyrosine (Phe/Tyr) catabolic pathway (left) and the concentrations of SA in parental and *GSTZ1*-KO HepG2 cells (right). Data are shown as mean ± SEM (*n* = 6 in each group). (**B**) Western blot shows protein expression of HIF-1α and VEGFA in HepG2 cells and Huh7 cells treated with SA (0, 100, 200, 300, 500 μM) under normoxia for 48 hours. (**C**) Western blot shows cytoplasmic and nuclear protein expression of HIF-1α in HepG2 cells treated with SA (300 μM) for 48 hours. (**D**) Western blot shows protein expression of HIF-1α in HepG2 cells treated first with Phe (2.0 mM) or SA (300 μM) for 48 hours, then with NTBC (0, 4, 7, 14, 28 μg/mL) for the last 12 hours. (**E**) Western blot shows protein expression of HIF-1α and hydroxylated HIF-1α in *GSTZ1*-KO HepG2 cells treated with or without NTBC. (**F**) Western blot shows protein expression of HIF-1α and hydroxylated HIF-1α in *GSTZ1*-OE Huh7 cells treated with SA (300 μM) for 48 hours. (**G** and **H**) Migration by Transwell assays (scale bar = 10 μm). Data are shown as mean ± SEM (*n* = 3 in each group). (**I** and **J**) Tube formation assay. The original magnification of **I** and **J** is 10×. Data are shown as mean ± SEM (*n* = 3 in each group). Statistical analysis was performed using 2-tailed unpaired Student’s *t* test (**A**) or 1-way ANOVA with Tukey’s test (**G**–**J**); *P < 0.05, **P < 0.01.

Our previous finding has shown that the concentration of SA, analyzed by liquid chromatography/mass spectrometry (LC/MS), was 4.5-fold higher in *Gstz1^–/–^* mouse livers than that in WT mouse livers (22). Given that the metabolite SA is structurally similar to α-KG, an important regulator of the PHD2/HIF-1α axis, we assumed that accumulated SA may be responsible for activating the HIF-1α signaling pathway in *GSTZ1*-deficient hepatoma cells. First, we measured metabolite levels by LC/MS in *GSTZ1*-KO HepG2 cells treated with Phe and found that in *GSTZ1*-KO HepG2 cells, the concentrations of SA were higher than the parental cells (3.445 ± 0.46 μmol/L vs. 0.95 ± 0.56 μmol/L, *P* < 0.001) (Figure 5A). Next, we discovered that SA loading increased the protein levels of HIF-1α and VEGFA in HepG2 and Huh7 cells in a dose-dependent manner, under both normoxia and hypoxia (Figure 5B and Supplemental Figure 5A). Immunoblotting showed that the expression of nuclear HIF-1α protein increased in HepG2 cells in the presence of SA (Figure 5C). Interestingly, an inhibitor of 4-hydroxyphenylpyruvate dioxygenase 2-(2-nitro-4-trifluoromethylbenzoyl)-1,3 cyclohexanedione (NTBC), which blocks the production of homogentisate and downstream metabolites, could reverse the Phe-mediated upregulation of HIF-1α in HepG2 cells. However, it failed to inhibit HIF-1α expression induced by the downstream metabolite SA overloading (Figure 5D). Furthermore, NTBC decreased HIF-1α levels in *GSTZ1*-KO HepG2 cells (Figure 5E). Meanwhile, SA restored the reduction of HIF-1α caused by *GSTZ1* overexpression (Figure 5F). To determine the functional relevance of our findings, HUVECs and HAOECs were used in Transwell, migration, and tube formation assays, with CM harvested from HepG2-KO cells treated with or without NTBC or CM from SA-treated *GSTZ1*-OE cells. NTBC decreased *GSTZ1* depletion–induced tumor-associated angiogenesis, which was significantly restored by SA treatment (Figure 5, G–J, and Supplemental Figure 5, B–E). Taken together, the loss of GSTZ1 results in SA accumulation, which promotes HIF-1α stabilization.

### SA stabilizes HIF-1α by inhibiting PHD2-mediated hydroxylation in GSTZ1-deficient HCC cells.

**Figure 6:**
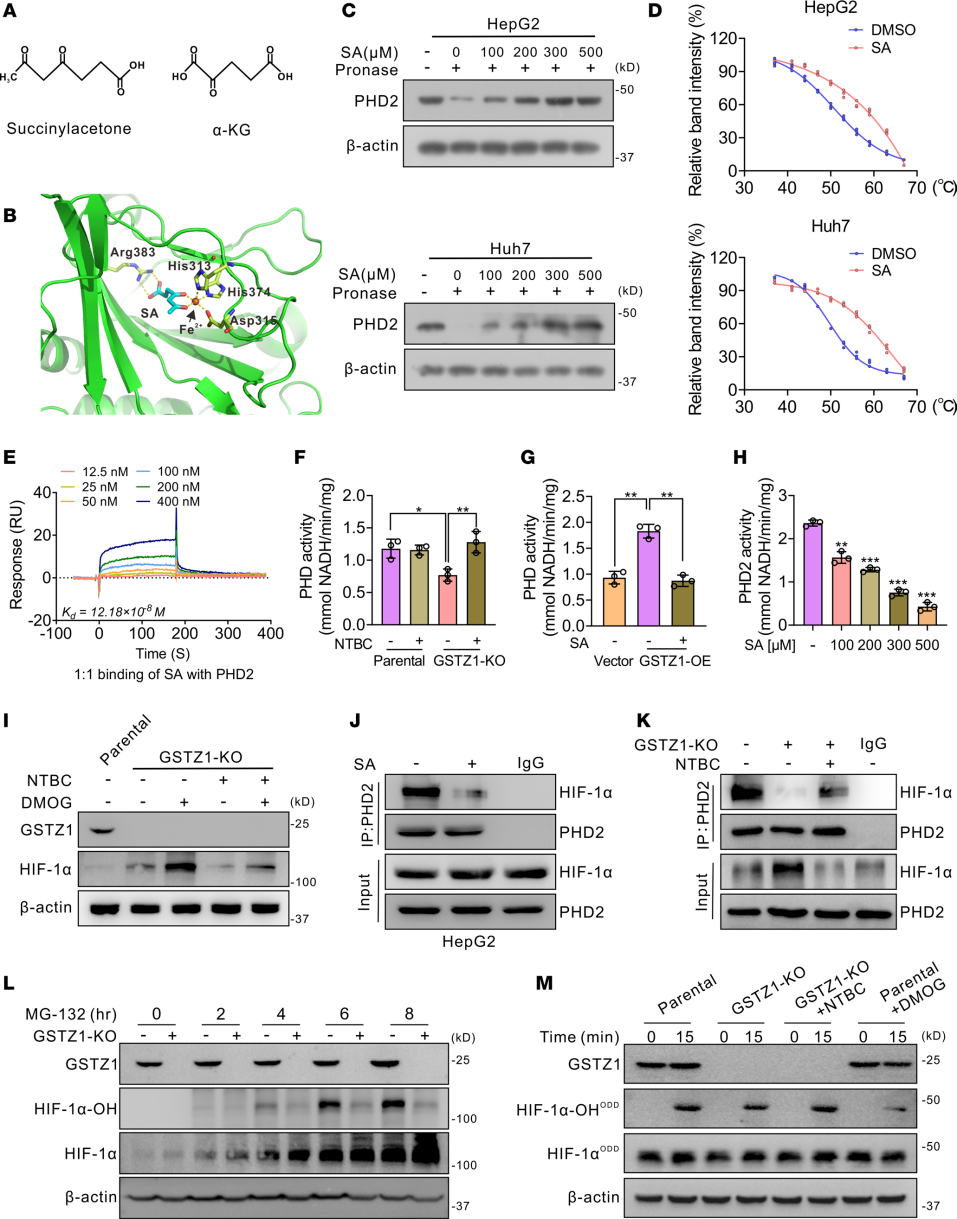
Lack of GSTZ1 impairs PHD2-mediated HIF-1α degradation by SA accumulation. (**A**) Schematic illustration showing SA is a structural analog of α-KG. (**B**) Geometry of SA with active PHD2 state and key residues. (**C**) DARTS assays for identification of direct binding between SA and PHD2 in HepG2 and Huh7 cells. (**D**) CETSA shows the binding affinity of SA to PHD2 in HepG2 and Huh7 cells. The data are presented as means ± SEM from 3 independent experiments. (**E**) SPR analysis of the binding between recombinant PHD2 with SA at the indicated concentrations. (**F** and **G**) In vitro PHD2 activity assay is performed in *GSTZ1*-KO HepG2 cells treated with or without NTBC and in *GSTZ1*-OE Huh7 cells treated with SA. Data are shown as mean ± SEM (*n* = 3 in each group). (**H**) In vitro PHD2 activity assay is performed by mixing bacteria-purified recombinant His-PHD2 with increasing amounts of SA. Data are shown as mean ± SEM (*n* = 3 in each group). (**I**) Immunoblots of parental or *GSTZ1*-KO HepG2 cells cultured with NTBC or 1 mM DMOG as indicated. (**J** and **K**) Co-IP assays to detect the direct interaction between PHD2 and HIF-1α treated with SA in HepG2 cells and in *GSTZ1*-KO HepG2 cells treated with or without NTBC. (**L**) Hydroxylated and total HIF-1α are detected from 0 to 8 hours after proteasomal blockade using 10 μM MG-132 in G*STZ1*-KO and parental HepG2 cells. (**M**) In vitro prolyl hydroxylation of the purified HIF-1α–ODD protein at 0 minute and 15 minutes using lysates from *GSTZ1*-KO or parental HepG2 cells, incubated for 24 hours with or without NTBC. Hydroxylation of HIF-1α–ODD is determined using the hydroxyprolyl-specific antibody at proline 564 (HIF-1α–OH-ODD), and total HIF-1α is detected (HIF-1α–ODD). DMOG-treated lysates are used as a negative control. Statistical analysis was performed using 1-way ANOVA with Tukey’s test; *P < 0.05, **P < 0.01, ***P < 0.001. NTBC, 2-(2-nitro-4-trifluoromethylbenzoyl)-1,3-cyclohexanedione.

It is well known that PHDs use α-KG as cosubstrate to hydroxylate target proteins on specific proline residues (27). Given the structural similarity between SA and α-KG (Figure 6A), we hypothesized that *GSTZ1* deficiency–mediated SA accumulation may stabilize HIF-1α by targeting PHD2, the most abundant form of PHD. To test this hypothesis, the AutoDock Vina software was used to predict the binding of SA to the α-KG binding sites of PHD2 (Figure 6B). Next, we conducted drug affinity responsive target stability (DARTS), cellular thermal shift assay (CETSA), and surface plasmon resonance (SPR) in HepG2 and Huh7 cells. Similar to the positive control α-KG, there was a direct binding between PHD2 protein and SA (Figure 6, C–E, and Supplemental Figure 6, A and B). To further examine the effect of SA on PHD2 in HCC cells more directly, we detected the protein expression levels of HIF-1α in HepG2 cells treated with SA and α-KG. The results showed that SA increased HIF-1α and VEGFA protein levels in a dose-dependent manner, while α-KG inhibited the effect of SA (Supplemental Figure 6, C and D). Moreover, SA level (3.45 ± 0.46 μmol/L) reached a comparative level as α-KG (1.98 ± 0.16 μmol/L) in *GSTZ1*-KO HepG2 cells (Supplemental Figure 6E). These data verified that SA stabilized HIF-1α by antagonizing the binding of α-KG to PHD2. Although PHD2 protein levels did not change in *GSTZ1*-KO HepG2 cells treated with or without NTBC (Supplemental Figure 6F), in vitro PHD2 activity assays revealed that *GSTZ1* deficiency suppressed PHD2 enzymatic activity, which was notably restored by NTBC treatment (Figure 6, F and G). GSTZ1 enhanced, but SA inhibited, the hydroxylation activity of PHD2 in a dose-dependent manner (Figure 6H). Consistently, depletion of *GSTZ1* remarkably increased levels of HIF-1α induced by dimethyloxalylglycine (DMOG), which is a potent inhibitor of PHD2, and NTBC partly decreased HIF-1α stabilization caused by *GSTZ1* deficiency in the presence of DMOG (Figure 6I). These results suggest that SA accumulation stabilizes HIF-1α by inhibiting PHD2 enzyme activity rather than by altering PHD2 protein levels. Furthermore, the interaction between HIF-1α and PHD2 was decreased after treatment with SA (Figure 6J and Supplemental Figure 6, G and H). Consistently, the interaction between HIF-1α and PHD2 was diminished in *GSTZ1*-KO cells and vice versa (Supplemental Figure 6I), whereas NTBC treatment partially restored this interaction (Figure 6K), suggesting that SA accumulation caused by *GSTZ1* deficiency may interfere with the interaction between HIF-1α and PHD2.

Considering that proline-hydroxylated HIF-1α is required for HIF-1α destabilization (28), we detected hydroxylated HIF-1α and total HIF-1α in *GSTZ1*-KO HepG2 cells. After treatment with the proteasome inhibitor MG-132, the total HIF-1α accumulated over time, but the hydroxylated HIF-1α did not change distinctly in *GSTZ1*-KO cells (Figure 6L). We then performed an in vitro prolyl hydroxylation assay to further determine PHD2 activity toward HIF-1α–oxygen-dependent degradation domain (HIF-1α–ODD) in *GSTZ1*-KO cells (29). As expected, the hydroxylated HIF-1α–ODD protein was decreased in *GSTZ1*-KO HepG2 cells, which was partially restored by NTBC treatment (Figure 6M). Furthermore, in vitro prolyl hydroxylation experiments revealed that SA could inhibit the hydroxylated HIF-1α–ODD protein (Supplemental Figure 6J). In addition, NTBC treatment did not effectively repress the malignant progression of tumors in either the DEN-induced WT group or the *Gstz1* gene–knockout mice (Supplemental Figure 6, K–M). Collectively, these experiments demonstrated that *GSTZ1* depletion induces SA accumulation, which binds to and inhibits the activity of PHD2, thereby preventing prolyl hydroxylation and subsequent proteasomal degradation of HIF-1α.

### Targeting HIF-1α and PD-L1 prevents HCC angiogenesis and progression in Gstz1^–/–^ mice.

**Figure 7:**
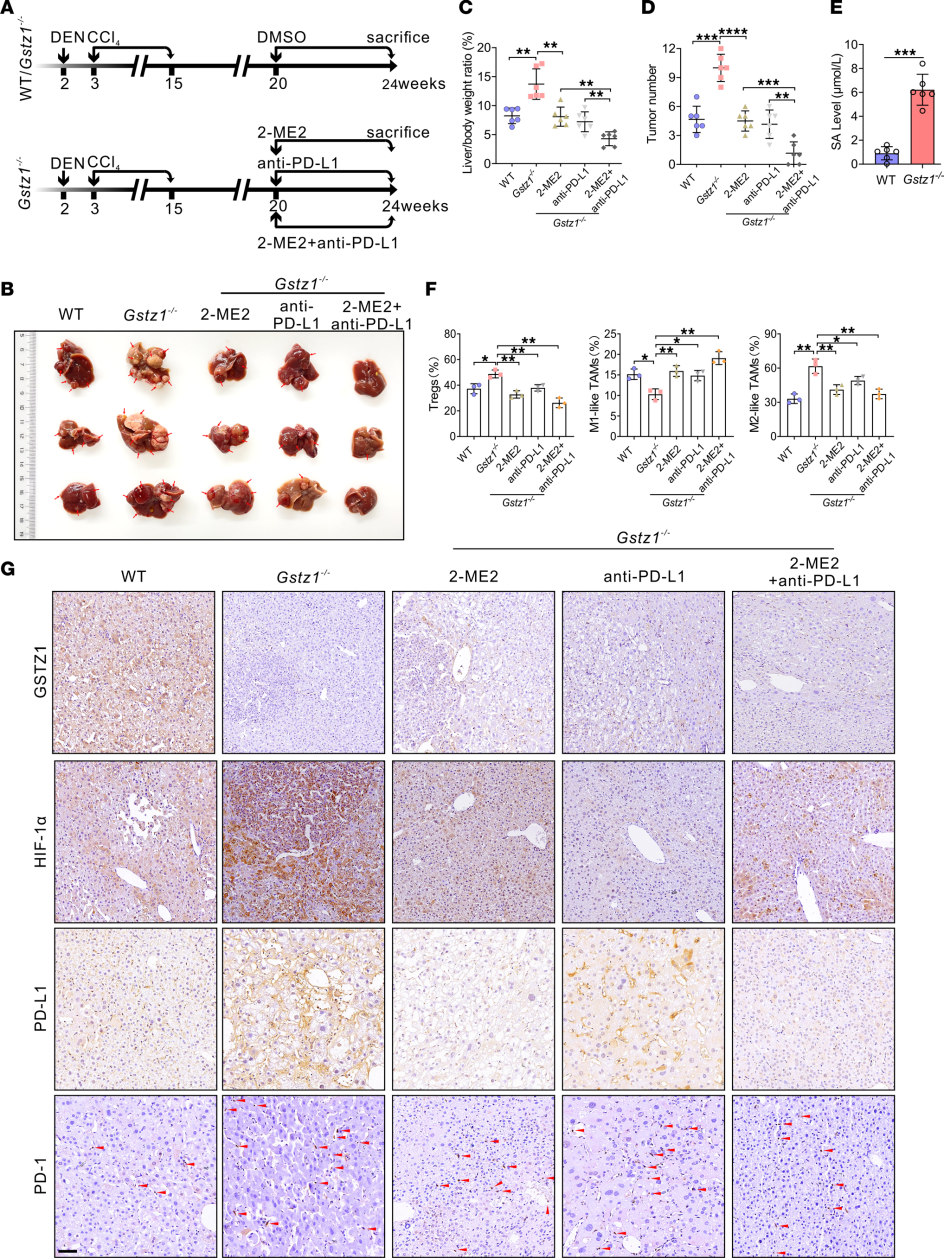
Targeting HIF-1α and PD-L1 prevents HCC growth and improves survival in *Gstz1^–/–^* mice. (**A**) Schematic representation of the experimental design and treatment schedule for in vivo studies. (**B**) Gross appearance of liver tumors. The red arrows represent nodules of the primary tumor. (**C** and **D**) Liver-to-body weight ratio (**C**) and tumor number (**D**). Data are shown as mean ± SEM (*n* = 6 mice per group). (**E**) The concentrations of SA in WT and *Gstz1^–/–^* mouse HCC tissues were measured using mass spectrometry. Data are shown as mean ± SEM (*n* = 6 mice per group). (**F**) The FOXP3^+^ Tregs and M1/M2-like TAMs in the liver tumors of mice. (**G**) Representative IHC (scale bar = 50 μm) images of GSTZ1, HIF-1α, PD-L1, and PD-1 in hepatic tumors. Red arrows designate cells staining positive for PD-1. Statistical analysis was performed using 1-way ANOVA with Tukey’s test (**C**, **D**, and **F**) or 2-tailed unpaired Student’s *t* test (**E**); *P < 0.05, **P < 0.01, ***P < 0.001, ****P < 0.0001. WT, wild-type; DEN, diethylnitrosamine; CCl_4_, carbon tetrachloride; 2-ME2, 2-methoxyestradiol.

Immune checkpoint blockade and inhibition of angiogenesis have synergistic effects in cancer treatment. To further explore whether *GSTZ1* deficiency contributes to HCC angiogenesis through HIF-1α activation, and whether combined targeting of HIF-1α and PD-L1 can improve the antiangiogenesis efficacy of HCC in vivo, we used the DEN/CCl_4_-induced HCC mouse model. *Gstz1^–/–^* mice were treated with DMSO control, anti–PD-L1, HIF-1α inhibitor 2-ME2, or a combination of anti–PD-L1 and 2-ME2, until the study endpoint (Figure 7A). *Gstz1^–/–^* mice exhibited liver tumorigenesis with an increased number of tumor masses and tumor nodules (Figure 7B). The combination of anti–PD-L1 and 2-ME2 inhibited tumor growth compared with either controls or each of these agents alone (Figure 7, C and D). In addition, in HCC tissue samples of *Gstz1^–/–^* mice, the concentrations of SA were significantly higher than the WT controls (6.53 ± 1.15 μmol/L vs. 1.20 ± 0.66 μmol/L, *P* < 0.001) (Figure 7E). There was no significant difference between α-KG in the 2 groups (Supplemental Figure 7A). In addition, we evaluated the prevalence of tumor-infiltrating FOXP3^+^ Tregs within CD4^+^ T cells and tumor-associated macrophages (TAMs) by flow cytometry. The results showed that the populations of FOXP3^+^ Tregs and M2-like TAMs (CD45^+^F4/80^+^CD11b^+^CD206^+^ cells) in *Gstz1^–/–^* mice were substantially higher than that of WT group, while the population of M1-like TAMs (CD45^+^F4/80^+^CD11b^+^CD86^+^ cells) was reduced in *Gstz1^–/–^* mice. Importantly, the administration of anti–PD-L1, HIF-1α inhibitor 2-ME2, or a combination of both therapies was able to reverse the prevalence of FOXP3^+^ Tregs and M2-like TAMs (Figure 7F and Supplemental Figure 7B). Immunoblot assay and IHC staining indicated that protein expression of HIF-1α, VEGFA, MMP2, MMP9, PD-1, and PD-L1, and of CD31-labeled microvessels, were increased in liver tumors in *Gstz1^–/–^* mice. In contrast, these protein indicators were decreased in mice treated with 2-ME2, anti–PD-L1, and the anti–PD-L1 and 2-ME2 combination. Importantly, in the combination treatment group, MVD was decreased compared with 2-ME2 or anti–PD-L1 alone (Figure 7G and Supplemental Figure 8, A and B). Thus, these data provide preclinical evidence for the use of inhibitors of HIF-1α plus PD-L1 inhibitor treatment to restrain tumor angiogenesis and reduce HCC progression.

### Clinical evidence that GSTZ1/SA/HIF-1α is activated in tumors from patients with HCC.

**Figure 8:**
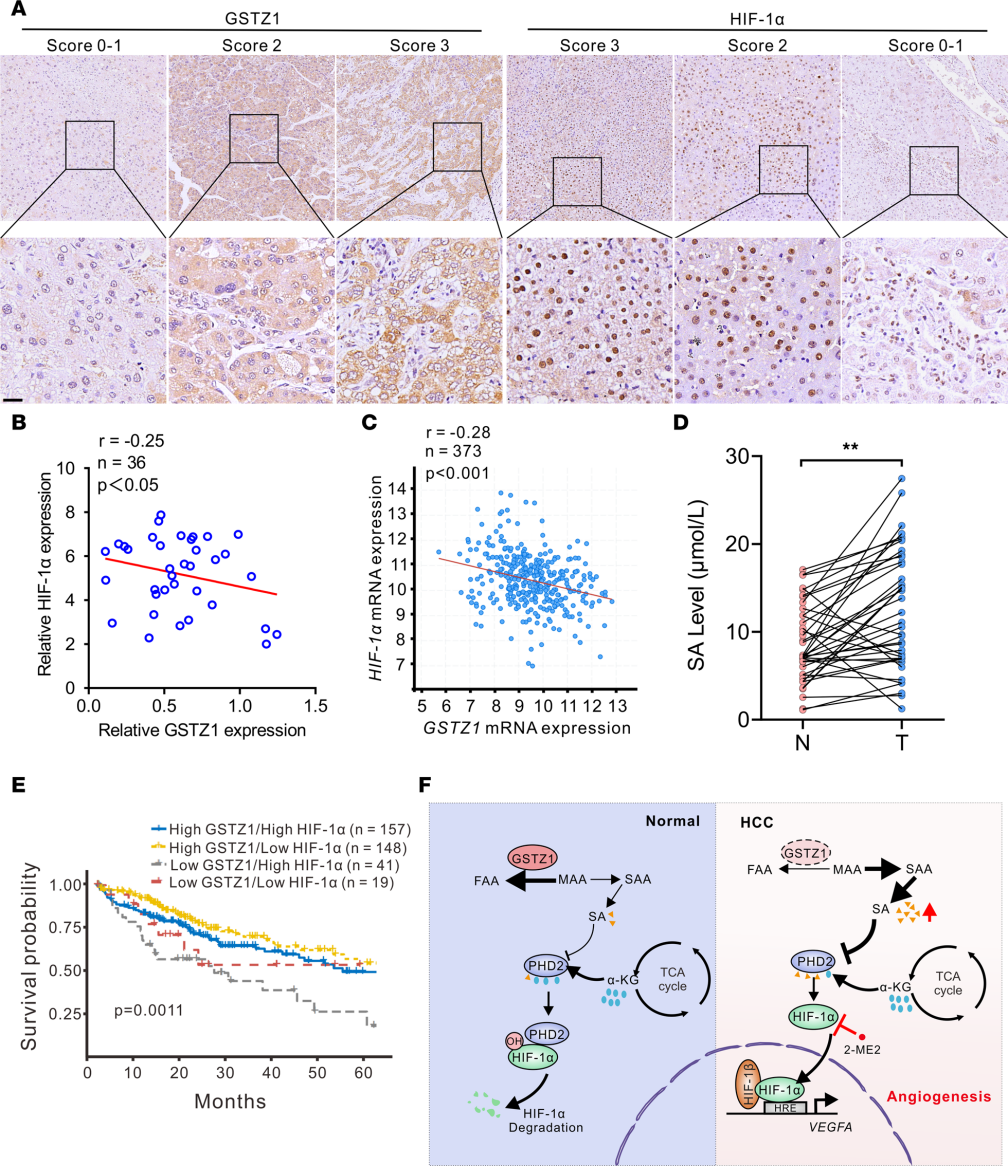
Clinical evidence that GSTZ1/SA/HIF-1α is activated in tumors isolated from patients with HCC. (**A**) Representative images of IHC staining of GSTZ1 and HIF-1α in HCC tissue. Scores are calculated based on the intensity and percentage of the stained cells (scale bar = 20 μm). The original magnification of the top row is 10×. (**B**) GSTZ1 and HIF-1α protein expression in 36 HCC and paired nontumor tissues. (**C**) Correlation analysis of *GSTZ1* and *HIF-1*α mRNA expression levels is conducted using data from 373 patients with HCC included in TCGA LIHC data set. (**D**) Relative SA levels of metabolites in HCC and paired nontumor tissues as measured using mass spectrometry. Data are shown as mean ± SEM (*n* = 40 in each group). (**E**) Overall survival in HCC patients with high (> 25th percentile) or low (≤ 25th percentile) mRNA expression levels of *GSTZ1* and *HIF-1*α, based on TCGA data. (**F**) A proposed model of how SA/HIF-1α/VEGFA axis activation promotes angiogenesis in *GSTZ1*-deficient cells. Statistical analysis was performed using Pearson’s *r* test (**B** and **C**), 2-tailed unpaired Student’s *t* test (**D**), or Gehan-Breslow-Wilcoxon test (**E**); **P < 0.01. N, nontumor; T, tumor; TCGA, The Cancer Genome Atlas. MAA, maleylacetoacetate; FAA, fumarylacetoacetate; SAA, succinylacetoacetate; OH, hydroxyl; DMOG, dimethyloxalylglycine; 2-ME2, 2-methoxyestradiol; HRE, hypoxia-responsive element.

Finally, we sought to assess the potential clinical relevance of *GSTZ1* deficiency and HIF-1α activation in patients with HCC. We performed IHC staining in a tissue microarray of HCC patient samples purchased from Shanghai Ming Yi Biotech (catalog LVC1805). Representative images of HCC tissue immunostaining for GSTZ1 and HIF-1α are shown in Figure 8A. Patients with low GSTZ1 expression showed a higher expression of HIF-1α. The results suggested that there was a negative correlation between GSTZ1 and HIF-1α expression (*r* = –0.25, *P* < 0.05, *n* = 36; Figure 8B and Supplemental Figure 1D). Consistently, a negative correlation between the mRNA levels of *GSTZ1* and *HIF-1*α was observed in a large cohort of patients with HCC from TCGA data set (*r* = –0.28, *P* < 0.001, *n* = 373; Figure 8C). Moreover, SA concentrations in HCC tissues were higher than in matched adjacent normal tissues (Figure 8D). Kaplan-Meier survival curve analysis showed that patients with low expression of GSTZ1 and high expression of HIF-1α had the lowest overall survival (median survival, *P* = 0.0011, Figure 8E). In summary, low expression of GSTZ1 or abnormal accumulation of SA with high expression of HIF-1α might predict poor outcomes in patients with HCC.

## Discussion

Cancer cells reprogram their metabolic pathways to adapt to the TME with low glucose, low oxygen, and low pH. Tyrosine catabolism mainly occurs in the liver, which expresses the highest level of tyrosine catabolic enzymes among organs (30). Notably, studies have revealed that tyrosine catabolic enzymes are dysregulated in HCC (31, 32). However, the mechanism that underlies the regulation of the tyrosine catabolic enzymes involved in HCC tumorigenesis has not been clearly defined. Here, we investigated the mechanistic linkage between the tyrosine catabolic enzyme GSTZ1 and the PHD2/HIF-1α axis, which controls proangiogenic VEGFA expression and promotes HCC angiogenesis. We uncovered that SA accumulated in HCC due to *GSTZ1* deficiency. Specifically, SA binds to PHD2 and inhibits its activity, preventing its interaction with the ODD domain of HIF-1α, thereby enhancing HIF-1α stability and promoting tumor angiogenesis by upregulating VEGFA expression (Figure 8F). Importantly, we also provide clinical evidence verifying the negative link between GSTZ1 and HIF-1α in tumors from patients with HCC, which helps us better understand the mechanisms by which GSTZ1 suppresses HCC progression and metastasis.

PHDs are members of the α-KG–dependent dioxygenases, which include several chromatin-modifying enzymes, and are emerging as key mediators of metabolic control and of cell fate (33). The zinc finger of PHD2 is responsible for the prolyl hydroxylation of HIF-1α (34). Inhibition of dioxygenases, even in hyperoxia, can be induced using small molecule metabolites that are antagonistic, competitive α-KG analogs (14, 35–37). The regulation of dioxygenase activity by α-KG and oxygen renders dioxygenase enzymes responsive to both oxygen tension and metabolic intermediates (38). Accumulation of succinate and/or fumarate, the metabolites of the TCA cycle, inhibits the activity of PHD2, leading to HIF-1α stabilization (14, 17). Disruption of oxoglutarate dehydrogenase and lipoic acid synthase results in the accumulation of L-2-HG, which stabilizes HIF-1α by inhibiting PHD2 activity (29). In addition, depleting cystathionine synthase results in H**_2_**S production, which can persulfidate PHD2 and inhibit its activity (34). Consistently, we also observed that the loss of GSTZ1 activated HIF-1α due to SA accumulation. Because of its structural similarities with α-KG, SA competitively binds to PHD2 and inhibits α-KG–dependent PHD2 activity, thereby preventing HIF-1α ODD domain hydroxylation. Moreover, abnormal accumulation of SA was found to promote the proliferation, migration, and angiogenesis ability of ECs. As suggested in a recent review (39), the term “oncometabolites” refers to a class of small molecular compounds that are significantly increased in tumors and have defined mechanisms through which they participate in the process of malignant progression. Our study found marked accumulation of SA in *GSTZ1*-deficient HCC and demonstrated the role of abnormal accumulation of SA in controlling tumor angiogenesis and vessel abnormalities. SA may be described as a potential oncometabolite that might promote angiogenesis of HCC. The present study demonstrates the role of abnormal accumulation of SA in controlling tumor angiogenesis and vessel abnormalities in the TME. We uncovered a distinct mechanism of metabolite-mediated HIF-1α activation and established the critical role of SA-activated HIF-1α in HCC angiogenesis, highlighting the importance of metabolites in regulating α-KG–dependent dioxygenases in shaping cell fate. SA may be a new oncometabolite, and it will be interesting to determine whether *GSTZ1* deficiency and SA accumulation influence the enzymatic activity of other 2-OG–dependent dioxygenases, such as DNA methylhydroxylase TET enzymes and RNA demethylase FTO enzymes (40). Future studies are needed to explore whether SA plays a key role in regulating histone and nucleic acid demethylases.

Given our data showing that HIF-1α inhibition alleviates the tumor-promoting effect of *GSTZ1* deficiency on HCC angiogenesis, we propose that a therapeutic strategy that suppresses SA-activated HIF-1α may improve current therapies that have limited effect on abnormal vasculature. Recently, immunotherapy using immune checkpoint inhibitors (anti–PD-1 and anti–PD-L1) has shown promising results in patients with advanced HCC (41, 42). Antiangiogenic treatment can normalize the tumor vasculature and recruit tumor-infiltrating cytotoxic T cells, leading to enhanced antitumor immunity (43). A potential advantage of combining anti–PD-L1 antibody with anti-VEGF antibody in advanced-stage HCC has been proven (44). However, tumors often develop drug resistance to antitumor angiogenesis drugs targeting VEGF (i.e., bevacizumab) or small molecule inhibitors that target VEGF receptors during treatment, which prompts the need for more effective and reasonable combination therapies (45). A previous study showed that simultaneous blockade of PD-L1 and inhibition of HIF-1α may represent a novel approach for cancer immunotherapy (46). Moreover, the HIF-1α inhibitor PX-478, combined with anti–PD-L1 treatment, can reverse the immunosuppressive microenvironment in gliomas (47). A recent study showed that combining HIF inhibitor 32-134D with anti–PD-1 may represent a breakthrough therapy for HCC (48). HIF inhibition may impact tumor progression by directly blocking its tumor-promoting function in tumor cells and by modulating the tumor-enabling function of the immune TME, with the potential to improve responses to immune checkpoint blockade (49). In our study, we showed that the combined treatment using anti–PD-L1 and 2-ME2 induced an augmented antineoplastic potency in the *Gstz1*^–/–^ mouse models, and the combination treatment exerted a more pronounced antitumor effect, assessed in terms of both tumor growth and survival, than each monotherapy. This indicates that combination therapy with anti–PD-L1 and 2-ME2 may represent a novel approach for HCC therapy. As 2-ME2 lacks specificity, future studies should explore more selective HIF-1α inhibitors that may achieve the benefits of inhibiting metabolic changes as well as angiogenesis. Additionally, since most HCC tissue specimens we collected were from patients with HBV infection, combination therapy may have certain limitations in HCC patients with other etiologies; future studies using a large number of clinical samples need to be conducted to confirm the efficacy of this combination therapy.

In summary, we describe a regulation of the SA/PHD2/HIF-1α axis by the metabolic enzyme GSTZ1. Our work demonstrates that *GSTZ1* deficiency promotes HCC angiogenesis by stabilizing HIF-1α via SA accumulation. In addition, the combination of targeting HIF-1α and PD-L1 could limit tumorigenesis and progression in *Gstz1^–/–^* mice, thus providing a potential target for HCC therapy.

## Methods

### Cell culture.

The human hepatoma cell line HepG2 (ATCC) and HAOECs (ATCC; PCS-100-011) were obtained. Huh7 cells and HUVECs were obtained from the Cell Bank of the Chinese Academy of Sciences (Shanghai, China). *GSTZ1*-KO HepG2 cells were generated by lentiCRISPR system as described previously (22). A recombinant adenovirus expressing GSTZ1 (AdGSTZ1) was generated using the AdEasy system in Huh7 cells, which endogenously express low levels of GSTZ1, and were infected with AdGSTZ1 to establish the *GSTZ1*-OE cell model. An analogous adenovirus expressing green fluorescent protein was used as control. Cells were maintained in MEM (for HepG2; Hyclone) or DMEM (for Huh7 and HUVEC; Hyclone) supplemented with 10% FBS (Natocor) and 1% penicillin/streptomycin (Hyclone) in a 5% CO_2_ incubator at 37°C. For the hypoxic cultures, cells were cultured in a hypoxia chamber flushed with a humidified gas mixture of 1% O_2_, 5% CO_2_, and 95% N_2_ at 37°C for 12 hours.

### Patient specimens.

A total of 42 HCC and paired adjacent noncancerous tissues were obtained from The Second Affiliated Hospital of Chongqing Medical University between 2015 and 2021. Each sample was frozen immediately after surgery and stored in liquid nitrogen for later use. IHC staining was performed in a tissue microarray of HCC patient samples purchased from Shanghai Ming Yi Biotech (catalog LVC1805).

### TCGA data analysis.

*GSTZ1*, *VEGFA*, and *HIF-1α* mRNA expression profiles were obtained from TCGA LIHC data set. The Kaplan-Meier survival curves were generated with the survminer R package (Version 3.6.3).

### RNA-Seq analysis.

*GSTZ1*-KO HepG2 cells and parental cells were cultured under hypoxic conditions for 12 hours. Then, total RNA was extracted using TRIzol reagent (Invitrogen) according to the manufacturer’s instructions. RNA-Seq was performed at the Shanghai Novel Bio-Pharm Technology Co., Ltd, in Shanghai, China. Briefly, strand-specific RNA-Seq libraries were prepared using the NEBNext Ultra RNA Library Prep Kit for Illumina (New England Biolabs). Six samples were sequenced on a HiSeq 4000 sequencing platform (Illumina). A bioinformatics pipeline was used to analyze differential gene expression data between *GSTZ1*-KO and parental cells. The RNA-Seq data were submitted to the Gene Expression Omnibus database under the accession number GSE192760.

### IHC and immunofluorescence staining.

GSTZ1, VEGFA, HIF-1α, PD-L1, PD-1, and CD31 were detected by IHC as previously described (50). In brief, liver tissue samples from paraffin-embedded human or mouse tumors were incubated at 4°C overnight with dilutions of the indicated primary antibodies (anti-VEGFA, ab1316, Abcam; anti–HIF-1α, ab51608, Abcam; anti–PD-L1, 13684, Cell Signaling Technology; anti–mPD-1, AF1021, R&D Systems, Bio-Techne; anti-CD31, 77699, Cell Signaling Technology). The slides were then incubated with a secondary anti-rabbit or anti-mouse IgG (ZSGB-BIO) and visualized using 3,3′-diaminobenzidine (ZSGB-BIO). Stained slides were scanned using the Motic Easy Scanner. Images were acquired using DSA assistant Lite (Motic VM V1 Viewer 2.0).

For tissue immunofluorescence staining, freshly prepared frozen liver sections were blocked with 5% goat serum for 1 hour at 25°C, then incubated with anti-CD31 (at 1:200 dilution in PBS, ab222783, Abcam) overnight at 4°C. Next, bound primary antibody was detected using an Alexa Fluor 488–conjugated goat anti-mouse IgG (A-10680, Invitrogen), and the nuclei were stained with DAPI (Roche, 1:200). The samples were then mounted with Fluoromount-G (Southern Biotech), and immunofluorescence images were acquired using a confocal microscope (FV3000, Olympus). The images were further processed using ImageJ 1.49v (NIH) or OlyVIA VS200 (Olympus).

### siRNA and plasmid DNA transfection.

HepG2 cells plated in collagen-coated 100 mm dishes at a density of 1 × 10^6^ cells were incubated overnight in complete MEM. Cells were then washed once with MEM, then incubated with 4 mL of Opti-MEM (Gibco) containing transfection mixture: 25 μL of Oligofectamine (Invitrogen) and 25 μL of 10 μM siRNAs targeting HIF-1α in 600 μL of Opti-MEM. At 48 hours posttransfection, cells were harvested and assayed for immunoblotting. For plasmid DNA transfection, Huh7 cells were transfected with 5 μg of *GSTZ1* plasmid using Lipofectamine 3000 (Invitrogen) following the manufacturer’s protocol. At 36 hours posttransfection cells were cultured under hypoxic conditions for another 12 hours before harvest.

### qRT-PCR.

Total RNA was extracted from cell lines and frozen tumor specimens using TRIzol reagent (Invitrogen) according to the manufacturer’s instructions. Purified RNA samples were reverse-transcribed into cDNA using the PrimeScript RT Reagent Kit with gDNA Eraser (RR047A, Takara). Quantitative real-time PCR analysis of target genes was performed using the SYBR Green qPCR Master Mix (Bio-Rad) with specific primers (Supplemental Table 1). The qPCR reaction was performed in 10 μL of reaction mixture: 2 μL of cDNA, 0.5 μL each of 10 μM forward and reverse primers, 5 μL of iTaq Universal SYBR Green Supermix (Bio-Rad), and 2 μL of nuclease-free water. The reaction conditions were as follows: 1 cycle at 9°C for 30 seconds, followed by 35 cycles of amplification at 95°C for 10 seconds, at 62°C for 30 seconds, and then at 72°C for 30 seconds. The relative gene expression was calculated using the ΔΔCT method using β-actin as the reference gene for normalization. Each sample was analyzed in triplicate.

### Western blotting.

All procedures were performed as we previously reported (50). Cytoplasmic and nuclear proteins were extracted using the Nuclear and Cytoplasmic Protein Extraction Kit (Beyotime) in accordance with the manufacturer’s instructions. The steps are roughly as follows: cells were scraped off with cell scraper, washed with PBS, and collected by centrifugation at 100*g* for 2 minutes at 4°C. Then, according to the manufacturer’s instructions, cytoplasmic protein extraction reagents A containing PMSF and cytoplasmic protein extraction reagents B were added successively, and the supernatant absorbed after vortex centrifugation was the cytoplasmic protein. The nuclear protein extraction reagent containing PMSF was added to the precipitation, and the supernatant absorbed from the precipitation after vortex centrifugation was nuclear protein. Antibodies were used as follows: anti-GSTZ1 (GTX106109, GeneTex), anti-VEGFA (ab1316, Abcam), anti–HIF-1α (36169, Cell Signaling Technology), anti–Hydroxy–HIF-1α (3434, Cell Signaling Technology), anti–β-actin (TA-09, ZSGB-BIO), anti-GAPDH (AG019, Beyotime), anti-PHD2 (4835, Cell Signaling Technology), anti-MMP2 (GTX133806, GeneTex), and anti-MMP9 (S1241, Bioworld). The protein samples were separated by 10% SDS-PAGE and transferred to PVDF membranes (MilliporeSigma). The immunoblots then were probed with the indicated antibodies. Finally, protein bands were visualized with ultrasensitive chemiluminescence substrate kits (Biosharp).

### ELISA.

Cells were cultured under hypoxic conditions for 12 hours, and cell supernatants were collected for VEGFA detection. The amount of VEGFA in the CM was determined using the Human VEGF Quantikine ELISA Kit (Proteintech) by measuring absorbance values at 450 nm in accordance with the manufacturer’s instructions.

### Wound scratch assay.

HUVECs and HAOECs (10,000 cells/well) were cultured with CM from hypoxia-treated *GSTZ1*-KO HepG2 cells or *GSTZ1*-OE Huh7 cells until 90% confluent, followed by scratching using WoundMaker (Essen BioScience) to create wounds. After 24 hours, cells migrating at the front of the wound were photographed by the IncuCyte ZOOM Live-Cell Imaging system (Essen BioScience).

### Cell proliferation and Transwell assays.

HUVECs and HAOECs were seeded in 96-well plates at 2,000 cells/well, then cultured with CM from hypoxia-treated *GSTZ1*-KO HepG2 or *GSTZ1*-OE Huh7 cells. The plates were scanned by the IncuCyte ZOOM Live-Cell Imaging system (Essen BioScience), and phase-contrast images were acquired at 0, 24, 36, 48, and 60 hours. The final number of cells was equal to the number of cells seeded at day 0 divided by the area of cells seeded at day 0 and multiplied by the final area of cells. For the migration assays, 1 × 10^4^ HUVECs and HAOECs were seeded onto the upper chamber of a Transwell insert with serum-free DMEM medium, while the underside chamber of the Transwell insert was filled with CM derived from hypoxia-treated *GSTZ1*-KO HepG2 cells or *GSTZ1*-OE Huh7 cells. After 24 hours, the inserts were fixed with 0.5% crystal violet. Images were acquired under a microscope (IX73, Olympus).

### PHD2 activity assay.

For exogenous PHD2 enzyme activity, His-tagged pET28a PHD2 constructs were expressed in *Escherichia coli* BL21 (DE3), and their expression was induced with 1.0 mM isopropyl β-d-1-thiogalactopyranoside (IPTG). We purified cell lysates to obtain PHD2 protein and used it for enzyme activity assays. For endogenous PHD2 enzyme activity, we prepared cells in a T75 flask and added Reagent A, which homogenized the cells. A typical assay mixture containing 20 mM MES buffer pH 6.5, 2 mM ATP, 2 mM CoA, 10 mM MgCl_2_, and 1 mM succinate was incubated time dependently (for 0, 2, 4, 6, and 8 minutes) at 37°C. The assay mixture (100 μL) was then added to 100 μL of acidic colorimetric solution to stop the reaction, and the mixture was incubated at room temperature for 5 minutes. The enzymatic activity was measured by absorbance values at 340 nm according to the manufacturer’s instructions (AmyJet Scientific).

### Orthotopic HCC model in mice.

BALB/c nude mice (4 to 6 weeks old, male, 20–25 g) were used to construct the syngeneic orthotopic liver cancer models. Briefly, 1 × 10^6^ HepG2 cells with or without *GSTZ1* knockout were suspended in a 40 μL PBS/Matrigel (356234, BD Biosciences) mixture (1:1 v/v ratio) and implanted into the liver lobe. Four weeks later, tumor-bearing mice were intraperitoneally injected with 2-ME2 (MCE) at a dose of 50 mg/kg/d (twice a week) for 4 weeks. At 8 weeks postimplantation, mice were sacrificed, and liver tissues were harvested for histological examination.

### Endothelial cell tube formation assay.

Briefly, a 96-well plate coated with 100 μL of 1 mg/mL Matrigel (BD Biosciences) was incubated at 37°C for 30 minutes to polymerize. Then, 1 × 10^4^ HUVECs and HAOECs were seeded into each well of a precoated 96-well plate and incubated with CM. After 6 hours, capillary-like tubes were photographed (original magnification, 40×) from 4 randomly chosen fields, then analyzed with Image ProPlus 8.0 software.

### Aortic ring sprouting assay.

Aortas were excised from 8-week-old C57BL/6 mice. Aortic rings were embedded in 150 μL Matrigel (BD Biosciences) in a 24-well culture plate. CM was added to the wells in a final volume of 200 μL culture medium. The aortic rings were incubated at 37°C for 6 days with medium replaced with fresh medium every other day. On day 6, the microvessel sprouting was photographed and scored from 0 (least positive) to 5 (most positive) in a double-blinded manner; 3 independent experiments were carried out with 5 rings per group in each assay. Representative micrographs were shown.

### Co-IP.

Huh7 and HepG2 cells were plated into 10 cm dishes until 80% confluent. SA (50 μg/mL) was added to the medium for 24 hours. Cells were lysed with cell lysis buffer (Beyotime) containing a protease inhibitor cocktail (Roche). Protein concentrations were measured using a BCA protein assay (Dingguo). Equal protein samples were incubated with anti–HIF-1α (36169, Cell Signaling Technology) or anti-PHD2 (4835, Cell Signaling Technology) or anti-IgG (5946, Cell Signaling Technology) overnight at 4°C, followed by incubation with Protein G Agarose Beads (MilliporeSigma) at 4°C for 4 hours. The beads were lightly washed twice in cell lysis buffer, followed by 1 wash in PBS containing 0.1% Tween 20. The bound antigen was finally eluted and prepared for Western blotting analysis.

### Metabolite detection and analysis.

For cell samples, *GSTZ1*-KO HepG2 and parental cells were washed twice with PBS, and metabolites were extracted with 400 μL cold methanol and acetonitrile (1:1, v/v), followed by the addition of the 2-chloro-d-phenylalanine internal standard. The mixture was centrifuged for 20 minutes at 14,000*g* at 4°C, and then 10 μL of the supernatant was injected into an Agilent 1290 Infinity II LC System coupled to an Agilent 6495c mass spectrometer. The concentrations of metabolites were detected using LC-MS/MS analysis.

For tissue samples, tissue was homogenized with an internal standard (2-chloro-d-phenylalanine) using a tissue homogenizer, followed by metabolite extraction with a mixture of methanol/water (1:1, v/v). The samples were centrifuged for 20 minutes at 4°C and 14,000*g* to pellet insoluble material, and supernatants were transferred to clean tubes.

### PHD2 expression and purification.

His-tagged pET28a-PHD2 constructs were expressed in *E*. *coli* BL21 (DE3), and expression was induced with 0.2 mM IPTG. Cells were resuspended in a buffer containing 20 mM Tris (pH 8.0), 300 mM NaCl, and 10 mM imidazole, then lysed using sonication for 30 minutes. Cell lysates were purified using a Ni-NTA affinity column (GE Healthcare, now Cytiva). PHD2 was eluted in 20 mL of elution buffer (20 mM Tris-HCl pH 8.0, 300 mM NaCl, 400 mM imidazole).

### In vitro hydroxylation assay.

Prepared *GSTZ1*-KO HepG2 cells and control group cells were treated with or without NTBC in a 15 cm cell culture dish, and purified HIF-1α–ODD (530 to 826 residues) were purchased from Abcam (ab48734). The cellular extract was prepared in 1 mL of reaction buffer (20 mM HEPES pH 7.5, 5 mM KCl, and 1.5 mM MgCl_2_) followed by 2 freeze/thaw cycles in an ethanol/dry ice bath. The lysates were passed 8 times through a 21-gauge needle, followed by 2 passages through a 26-gauge needle before centrifugation (17,000*g* at 4°C for 30 minutes). The supernatants were aliquoted and stored at −80°C. The hydroxylation assay was performed by incubating 10 mM HIF-1α–ODD with 25 μL of *GSTZ1*-KO HepG2 cell extract in the reaction buffer for 15 minutes at 37°C. The reaction was stopped by the addition of SDS loading buffer, and the proteins were separated using SDS-PAGE electrophoresis. Hydroxylation was measured using an HIF prolyl hydroxylation–specific antibody (3434T, Cell Signaling Technology). DMOG was used as a negative control in this assay as an inhibitor of PHD2 activity. Measurements of DMOG of HIF-1α hydroxylation were performed in a similar manner, except that the lysate was preincubated with the compounds for 10 minutes at 37°C before the addition of the HIF-1α–ODD protein.

### DARTS.

DARTS was conducted to identify the potential targets of SA. Briefly, 50 × 10^6^ cells were lysed in M-PER (78501, Thermo Fisher Scientific) with a protease inhibitor cocktail and phosphatase inhibitor cocktail. TNC buffer (50 mM Tris-HCl pH 8.0, 50 mM NaCl, and 10 mM CaCl_2_) was added to the lysate, and the protein concentration was determined using BCA assay (Beyotime). Protein concentration was adjusted to 4 μg/μL cells, and the lysates were incubated with varying concentrations of either SA or DMSO (vehicle) for 1 hour at room temperature and digested with Pronase (1:2,000 for PHD2, TopScience) for 30 minutes at room temperature. The digestion was stopped by adding a protease inhibitor cocktail, and the samples were immediately placed on ice. Subsequently, Western blotting was used to determine whether PHD2 was a direct target of SA. GAPDH was used as a negative control.

### CETSA.

CETSA was performed to determine the direct binding between SA and PHD2 in cells. Briefly, 4 × 10^6^ HepG2 and Huh7 cells were pretreated with 300 μM SA for 12 hours before being subjected to the CETSA protocol. Cells were chilled on ice, washed with PBS buffer plus protease inhibitor cocktail, and then subjected to 3 freeze/thaw cycles using dry ice and Thermal Cycler to lyse cells. Then, the cells were centrifuged at 20,000*g* for 20 minutes at 4°C to separate between the lysate and cell debris with aggregated proteins. Next, the supernatant was transferred into 200 μL PCR tubes; all samples were heat shocked in a Bio-Rad T100 Thermal Cycler at the indicated temperature for 3 minutes to denature proteins, then immediately cooled down at room temperature for 3 minutes. Finally, the supernatant was boiled with 4× loading buffer for Western blotting. The bands were quantified using the Gel-Pro analyzer software (http://www.gelanalyzer.com/?i=1) and plotted with 3 biological replicates.

### SPR.

Affinity analysis was performed using a Biacore X100 instrument (GE Healthcare Life Sciences, now Cytiva). His-PHD2 protein (Sino Biological, 11084-H08H) was directly immobilized on the sensor chip NTA. Then, 20 μg/mL of PHD2 in immobilization buffer was injected into the Fc2 sample channel at a flow rate of 10 μL/min. The metabolite SA or α-KG was serially diluted with the running buffer to obtain concentrations of 400, 200, 100, 50, 25, and 12.5 nM, respectively. Different concentrations of metabolite were then injected into the Fc2-Fc1 channels at a flow rate of 30 μL/min, with a contact time of 120 seconds, followed by a dissociation time of 400 seconds. After each cycle of interaction analysis and analyte injection, the association and dissociation processes were all handled in the running buffer. Data analysis was performed on the Biacore X100 computer, using the Biacore X100 evaluation software.

### Flow cytometry.

For flow cytometry analysis of in vitro mouse immune cells, the mouse T cells were incubated with 1× Golgiplug (BD Biosciences) for 6 hours. Immune cells were stained with surface antibodies in PBS for 30 minutes as described previously (51). Fixation and permeabilization processes were carried out with fixation buffer (BD Biosciences) according to the manufacturer’s protocols. The immune cells were then stained with antibodies in PBS to detect proteins. The following antibodies were used for staining: Brilliant Violet 510 anti-mouse/human CD11b (BioLegend, catalog 101245), FITC anti-mouse F4/80 antibody (BioLegend, catalog 123107), PerCP/Cyanine5.5 anti-mouse CD4 antibody (BioLegend, catalog 100540), APC anti-mouse CD206 antibody (BioLegend, catalog 141708), Brilliant Violet 421 anti-mouse CD86 antibody (BioLegend, catalog 105032), and PE anti-mouse FoxP3 antibody (Elabscience, catalog E-AB-F1238D). The flow cytometry was run using FACSCelesta flow cytometer (BD Biosciences), and the results were analyzed with FlowJo V10.7.1.

### Gstz1^–/–^ mouse study.

Heterozygous C57-Gstz1*^tm1Jmfc^*/Cnbc mice (EM: 04481) were obtained from the European Mouse Mutant Archive and were crossed to breed WT and *Gstz1^–/–^* mice. For the DEN and CCl_4_ -induced mouse HCC model, WT and *Gstz1^–/–^* mice (at 2 weeks of age) were administered an intraperitoneal injection of DEN (MilliporeSigma) at a dose of 75 mg/kg. At 3 weeks of age, the mice were administrated with 10% CCl_4_ (Macklin) intraperitoneally at a dose of 2 mL/kg twice a week for 12 weeks and then received an intraperitoneal injection of DEN at a dose of 50 mg/kg. At 20 weeks of age, *Gstz1^–/–^* mice were intraperitoneally administered 2-ME2 (50 mg/kg, once per week, S1233, Selleck), anti–PD-L1 (100 μg/kg, once per week), both, or vehicle (5% DMSO) (*n* = 6 per group) for 4 weeks. Mice in the NTBC treatment group received continuous NTBC treatment (8 mg/L, S5325, Selleck) through their drinking water until sacrifice (52). Mice were sacrificed at 24 weeks of age, and liver tissues were harvested for histological examination.

### Statistics.

Graphical representation and statistical analyses included 2-tailed unpaired Student’s *t* test, 1-way ANOVA with Tukey’s test, Pearson’s correlation coefficient, Gehan-Breslow-Wilcoxon test, and 2-way ANOVA with Bonferroni’s test, calculated using GraphPad Prism 8. *P* values less than 0.05 were deemed significant. The experiments were not randomized, except that the mice were randomly grouped before treatments. Samples were allocated to their experimental groups according to their predetermined type, and allocation was not blinded during the experiments and outcome assessment. Statistical information is otherwise provided in the figure legends.

### Study approval.

Primary HCC tissue samples and paired adjacent normal tissue samples were obtained from The Second Affiliated Hospital of Chongqing Medical University between 2018 and 2022, with approval from the Institutional Review Board of Chongqing Medical University. Written informed consent in accordance with a protocol approved by The Second Affiliated Hospital of Chongqing Medical University (Chongqing, China) was obtained from all patients. All animal experiments were performed under the guidelines of the institutional Animal Care and Use Committee at Chongqing Medical University. All animal procedures were also approved by the Research Ethics Committee of Chongqing Medical University.

### Data availability.

All data needed to evaluate the conclusions in the paper are present in the paper or the supplemental materials. Expression profile data analyzed in this study were deposited in Gene Expression Omnibus at GSE192760. See complete unedited blots in the supplemental material. Values for all data points are available in the Supporting Data Values file.

## Author contributions

HL, JX, QG, RL, KW, and NT conceived and designed the study. HL, JX, QW, KW, and NT developed methodology. QW, HL, RL, FY, BC, and XL acquired data. QW, HL, FY, CC, BQ, QG, and KW performed analysis and interpretation of data (e.g., statistical analysis, biostatistics). HL, QW, QG, RL, KW, BQ, and NT performed writing, review, and/or revision of the manuscript. QW, LH, RL, QG, KW, BQ, and NT provided administrative, technical, or material support (i.e., reporting or organizing data, funding).

## Supplementary Material

Supplemental data

Supporting data values

## Version 1 10/31/2023

In-Press Preview

## Version 2 12/08/2023

Electronic publication
