# Supplementary material for: Signaling metabolite succinylacetone activates HIF-1α and promotes angiogenesis in GSTZ1-deficient hepatocellular carcinoma
Source: JCI Insight. 2023 Oct 31;8(23):e164968. doi: 10.1172/jci.insight.164968 (PMC10896004; doi:10.1172/jci.insight.164968)

Supplemental data

Signaling metabolite succinylacetone activates HIF-1α and promotes angiogenesis in *GSTZ1*-deficient hepatocellular carcinoma

Huating Luo*, Qiujie Wang*, Fan Yang*, Rui Liu*, Qingzhu Gao, Bin Cheng, Xue Lin, Luyi Huang, Chang Chen, Jin Xiang, Kai Wang#, Bo Qin# and Ni Tang#

Name	Accession Number	Source	Sequence (5'-3')
***VEGFA* (Human)**	NM_001025366.2	TsingKe Biological Technology	Forward: GAGGGCAGAATCATCACGAAG<br>Reverse: TGTGCTGTAGGAAGCTCATCTCTC
***PDGFA* (Human)**	NM_001278549.2	TsingKe Biological Technology	Forward: GCTGGTTTTGGTTATGGATTG<br>Reverse: GCCTCGTGGTTGGTGTTGTA
***IGF1R* (Human)**	NM_000146.4	TsingKe Biological Technology	Forward: ATGACATTCCTGGGCCAGTG<br>Reverse: TAGCTTGGCCCCTCCATACT
***FGFR* (Human)**	NM_023110.3	TsingKe Biological Technology	Forward: ACCACCTACTTCTCCGTCAATG<br>Reverse: CTTTTCTGGGGATGTCCAATAT
***MMP9* (Human)**	NM_004994.2	TsingKe Biological Technology	Forward: CGCCAGAACTACCACCAGG<br>Reverse: CGGCAAGTCTTCCGAGTAGTTT
***HIF-1α* (Human)**	NM_014585.6	TsingKe Biological Technology	Forward: ATGAAGTGTACCCTAACTAGCCG<br>Reverse: GTTCACAAATCAGCACCAAGC
***β-actin* (Human)**	NM_001101	TsingKe Biological Technology	Forward: AGGCCAACCGCGAGAAGATGACC<br>Reverse: GAAGTCCAGGGCGACGTAGCAC
***HIF-1α gRNA1* (Human)**	NM_014585.6	UBIGENE	Sequence: CAAGATGTGAGCTCACATTG
***HIF-1α gRNA2* (Human)**	NM_014585.6	UBIGENE	Sequence: AGCCCTAGATGGCTTTGTGA
***HIF-1α siNC* (Human)**	NM_014585.6	TsingKe Biological Technology	Forward: UUCUCCGAACGUGUCACGUTT<br>Reverse: ACGUGACACGUUCGGAGAATT
***HIF-1α siRNA1* (Human)**	NM_014585.6	TsingKe Biological Technology	Forward: GCCGAGGAAGAACUAUGAATT<br>Reverse: UUCAUAGUUCUUCCUCGGCTT
***HIF-1α siRNA2* (Human)**	NM_014585.6	TsingKe Biological Technology	Forward: GCUGAUUUGUGAACCCAUUTT<br>Reverse: AAUGGGUUCACAAAUCAGCTT

Supplemental Table 1. Primer sequences used in this study.

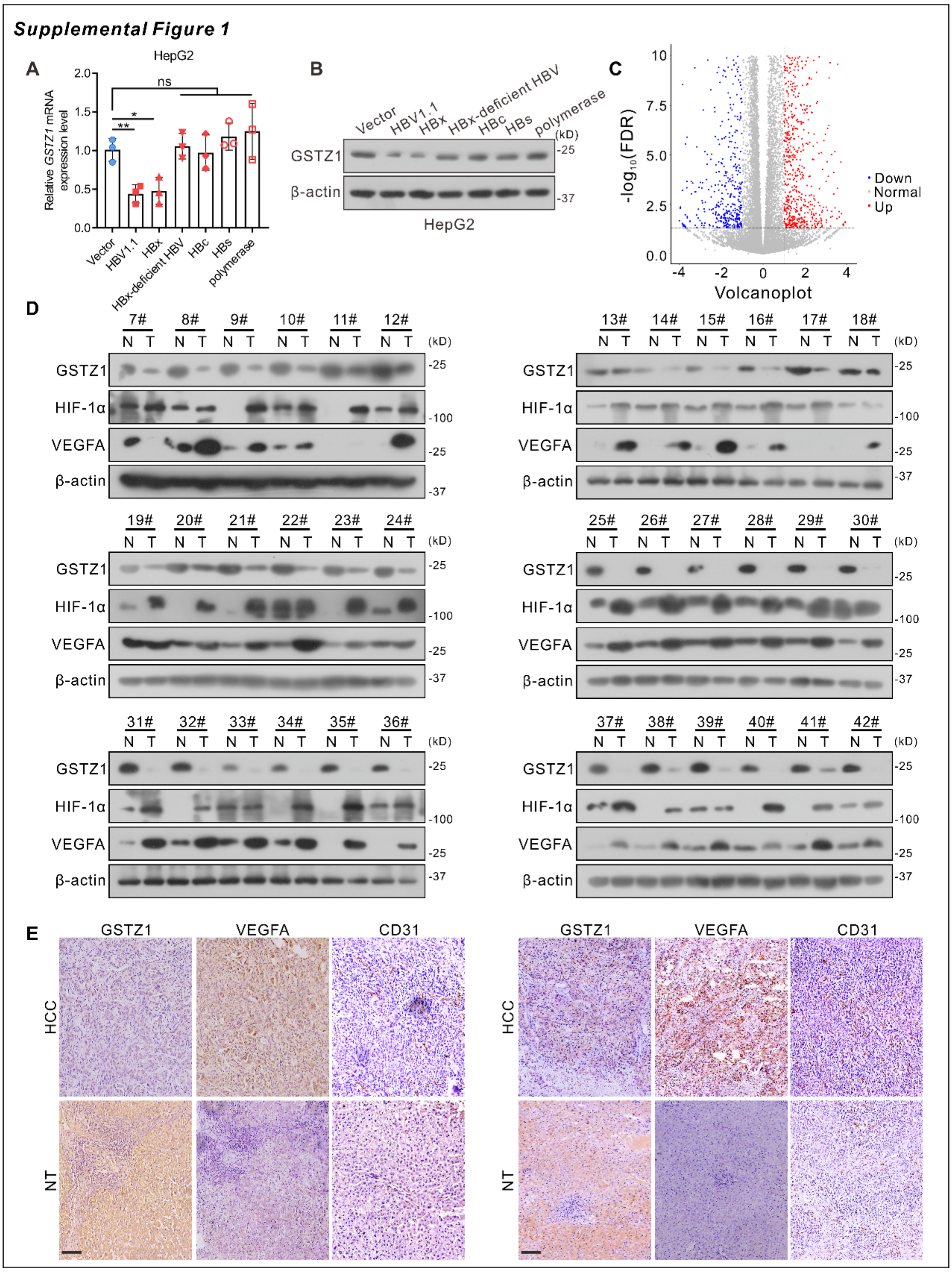

Supplemental Figure 1. GSTZ1 expression is negatively correlated with VEGFA in hepatoma cell lines and human HCC tissues. (**A**) The *GSTZ1* mRNA level in HepG2 cells analyzed by real-time qPCR. Data represent mean ± SEM for three independent experiments. (**B**) The protein level of GSTZ1 in HepG2 cells by western blotting. (**C**) Volcano plot of RNA-sequencing data obtained using *GSTZ1*-KO (*GSTZ1*-OE) HepG2 cells. (**D**)

GSTZ1, HIF-1α, and VEGFA protein expression in 42 HCC and paired non-tumor tissue specimens. (**E**) Representative GSTZ1, VEGFA, and CD31 IHC staining in three cases of HCC and paired non-tumor tissues (scale bar = 200 μm). Statistical analysis was performed using one-way ANOVA with Tukey’s test, $^{*}p < 0.05$, $^{**}p < 0.01$.

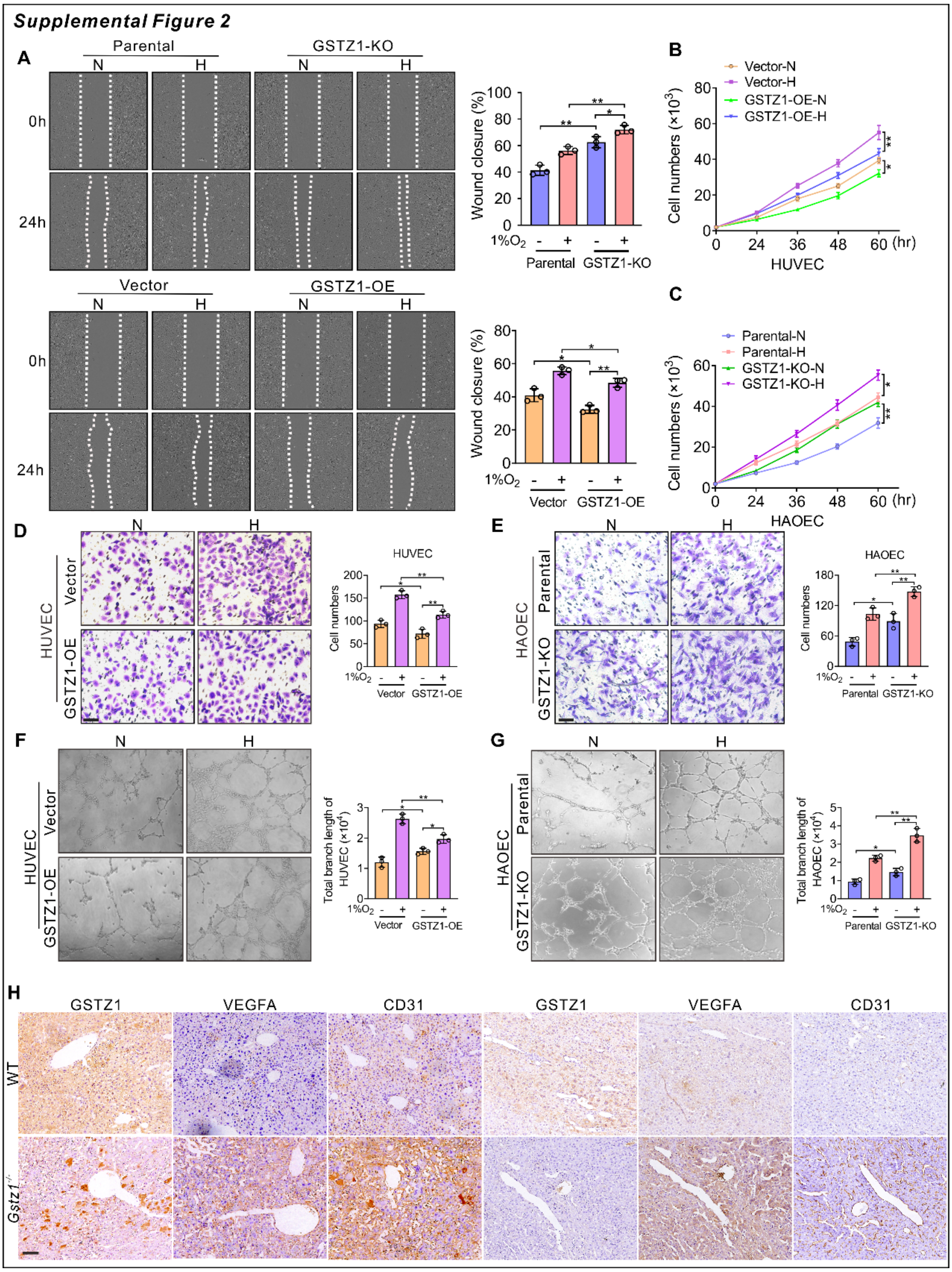

Supplemental Figure 2. GSTZ1 suppresses HCC angiogenesis in vitro and in vivo.(**A**) The migration of HUVECs treated with CM. (**B, C**) Cell growth curve. (**D, E**) Migration by transwell assays (scale bar = 10 μm). (**F, G**) Tube formation assays in HUVECs and HAOECs under CM. **H** Representative GSTZ1, VEGFA and CD31 IHC staining in WT group and *Gstz1*$^{-/-}$ mouse (scale bar = 200 μm). Data are shown as mean ± SEM (n = 3 in each group).

Statistical analysis was performed using one-way ANOVA with Tukey's test (A, D-G) or 2-way ANOVA with Bonferroni's test (B and C); *$p < 0.05$, **$p < 0.01$, ***$p < 0.001$. Abbreviations: N, Normoxia; H, Hypoxia; WT, wild-type.CM, conditional medium.

Supplemental Figure 3

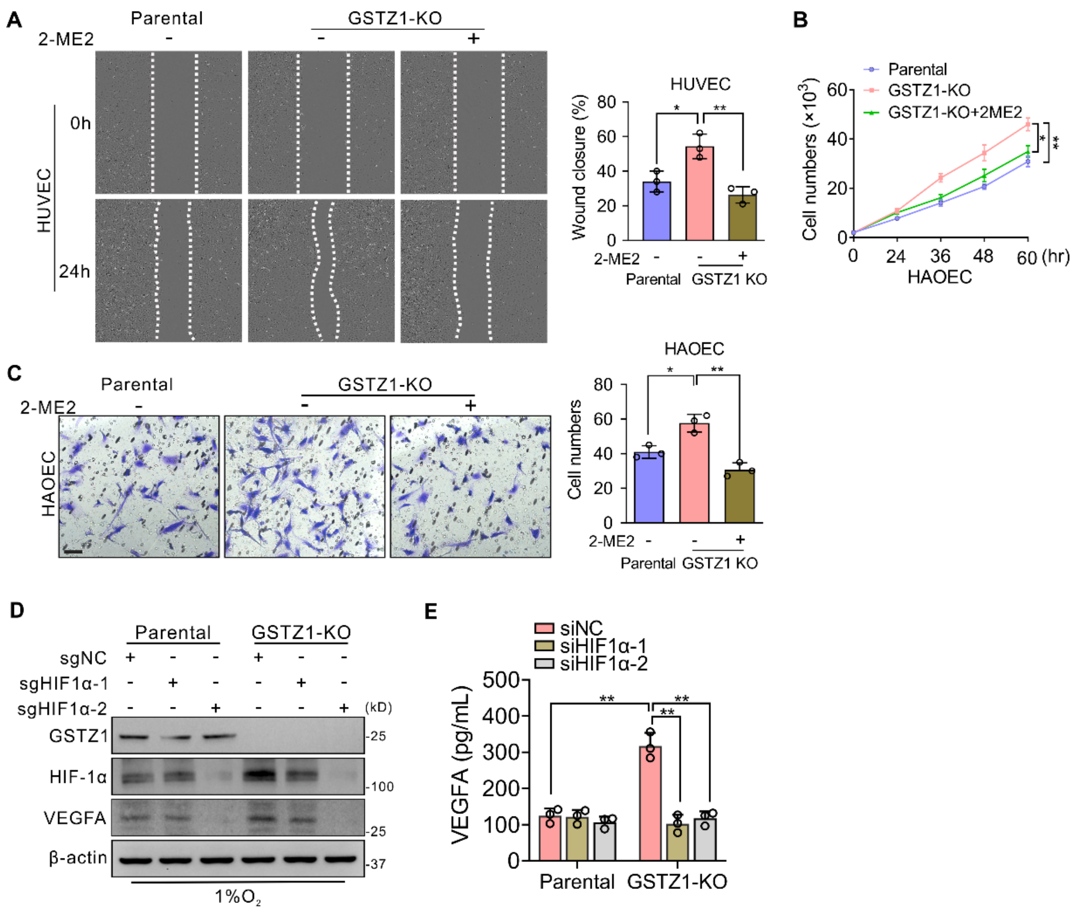

Supplemental Figure 3. GSTZ1 suppresses HCC angiogenesis by inactivating the HIF-1α signal pathway. (**A**) Migration of HUVECs treated with CM. (**B**) Cell growth curve. (**C**) Migration by transwell assays (scale bar = 10 μm). (**D**) Protein expression levels of HIF-1α, VEGFA and GSTZ1 in parental and *GSTZ1*-KO cells ectopically expressing sgNC or sgHIF-1α under hypoxia for 12 hr. (**E**) ELISA measurements of the VEGFA protein level in the

culture medium of *GSTZ1*-KO HepG2 cells under normoxia for 12 hr. Data are shown as mean ± SEM. Statistical analysis was performed using one-way ANOVA with Tukey's test (A, C and E) or 2-way ANOVA with Bonferroni's test (B); *$p < 0.05$, **$p < 0.01$, ***$p < 0.001$. Abbreviations: 2-ME2, 2-Methoxyestradiol; siNC, si-Negative Control; sgNC, sg-Negative Control.

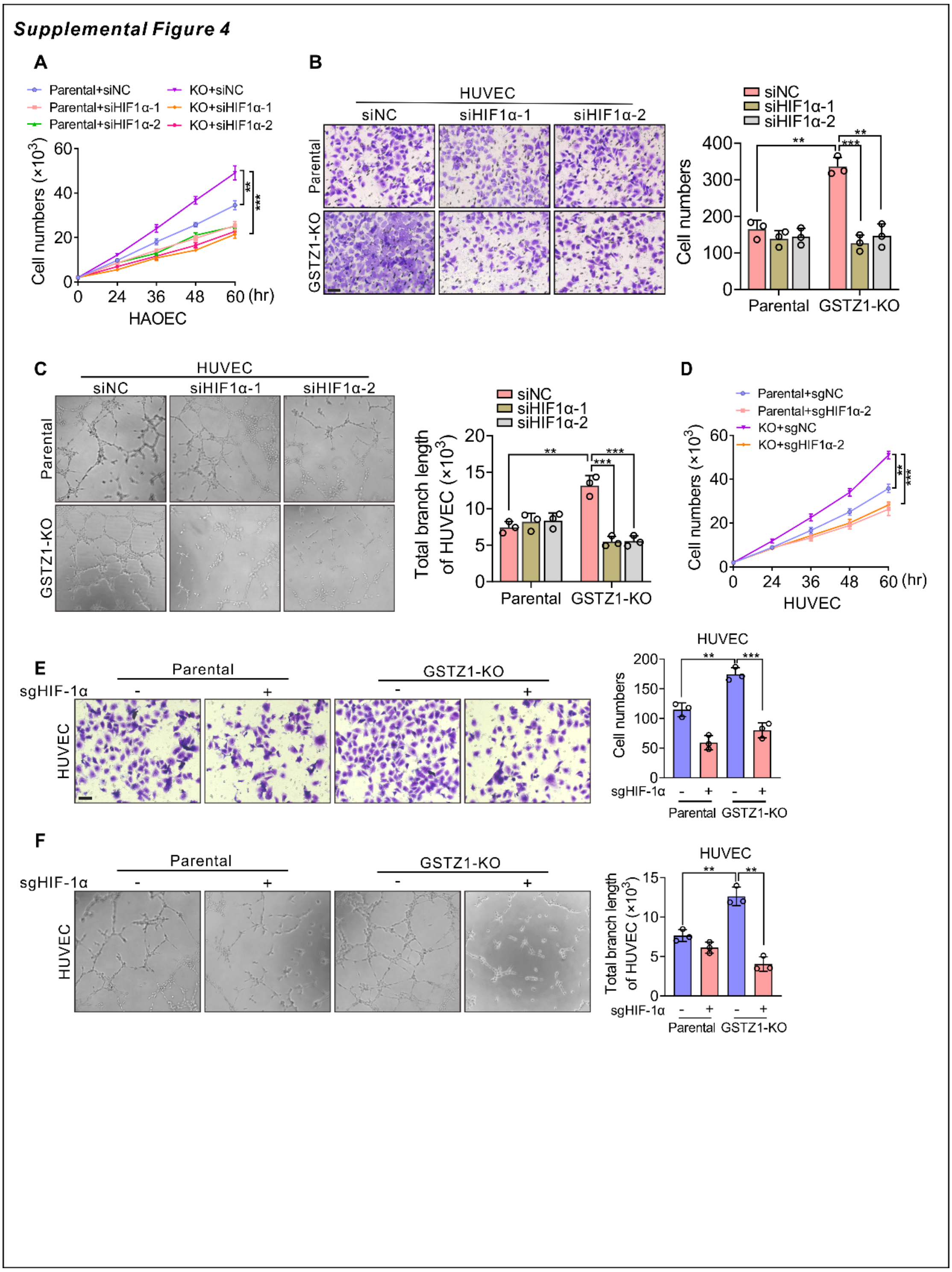

Supplemental Figure 4. HIF-1α inhibition reduced HUVEC and HAOEC proliferation, migration, and angiogenesis capacity caused by *GSTZ1* deficiency. (**A**) Cell growth curve. (**B**) Migration of HUVECs by transwell assays under normoxia for 12 hr (scale bar = 10 μm). (**C**) Tube formation assays in HUVECs under normoxia for 12 hr. (**D**) Cell growth curve. (**E**) The migration of HUVECs treated with CM under hypoxia for 12 hr. (**F**) Tube

formation assays in HUVECs under hypoxia for 12 hr. Values represent the mean ± SEM (n = 3 in each group). Statistical analysis was performed using 2-way ANOVA with Bonferroni's test (A and D) or one-way ANOVA with Tukey's test (B-C and E-F); ** $p < 0.01$, *** $p < 0.001$. Abbreviations: siNC, si-Negative Control; sgNC, sg-Negative Control.

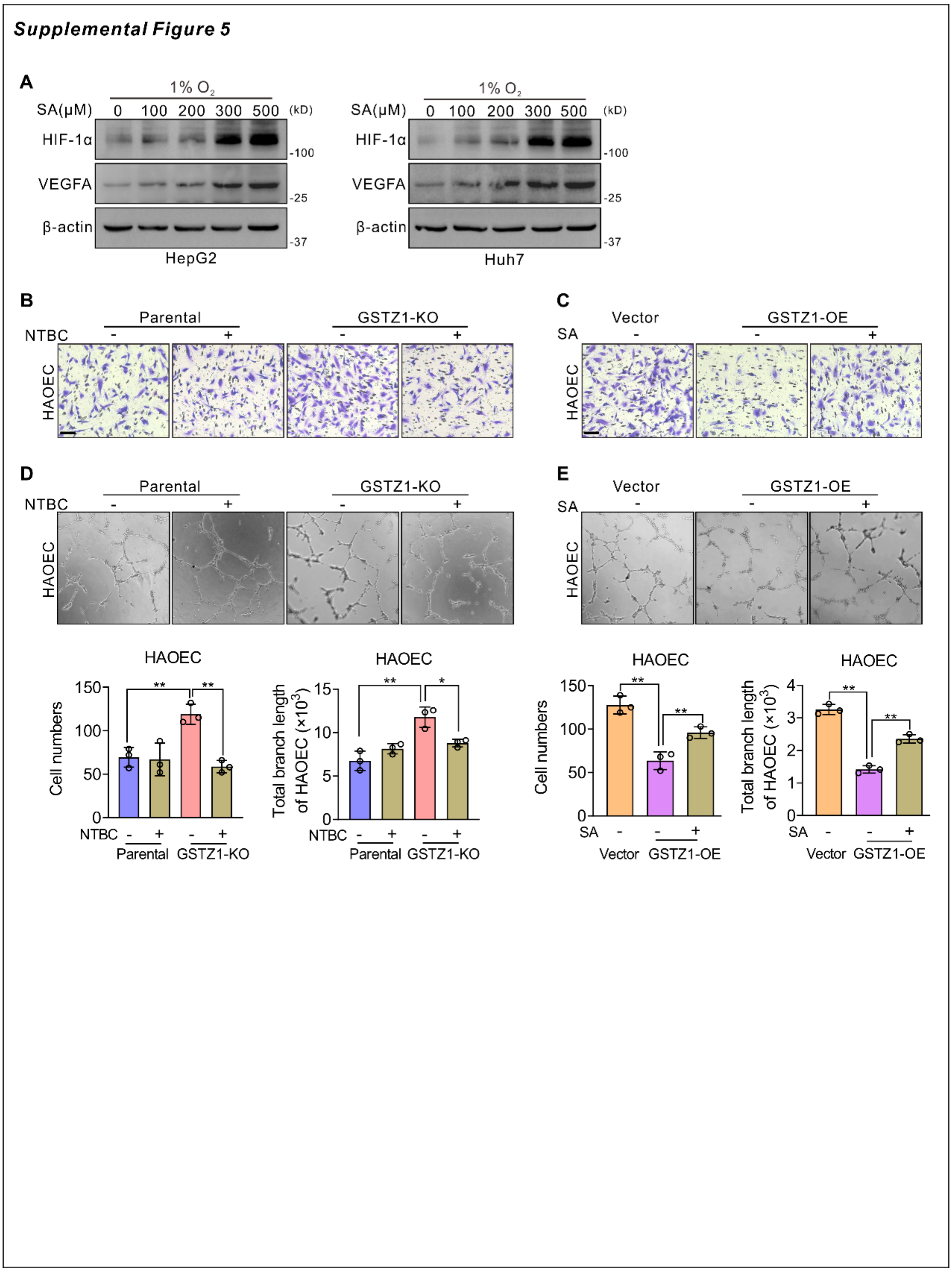

Supplemental Figure 5. Loss of GSTZ1 results in succinylacetone accumulation and HIF-1α activation. (**A**) Protein expression of HIF-1α and VEGFA in HepG2 and Huh7 cells treated with succinylacetone (SA, 0, 100, 200, 300 and 500 μM) under hypoxia. (**B, C**) Migration by transwell assays (scale bar = 10 μm). (**D, E**) Tube formation assays in HAOECs under CM. Values represent the mean ± SEM (n = 3 in each group). Statistical analysis

was performed using one-way ANOVA with Tukey's test; **p* < 0.05, ***p* < 0.01. Abbreviations: SA, succinylacetone; NTBC, 2-(2-nitro-4-trifluoromethylbenzoyl)-1,3 cyclohexanedione.

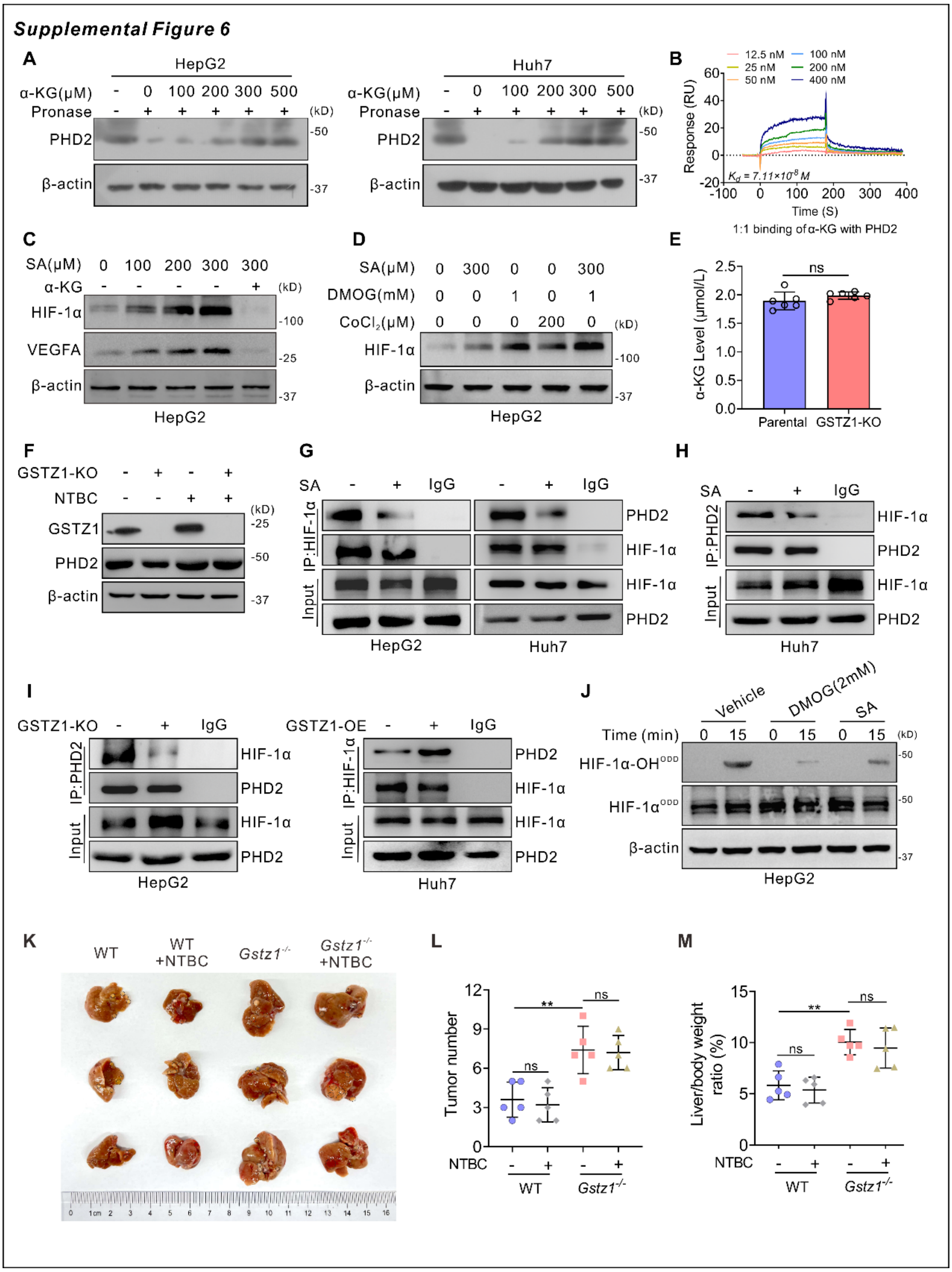

Supplemental Figure 6. Succinylacetone stabilizes HIF-1α by inhibiting PHD2-mediated hydroxylation in GSTZ1-deficient HCC cells. (**A**) DARTS assays for identification of the direct binding between α-KG and PHD2 in HepG2 and Huh7 cells. (**B**) SPR analysis of the binding between recombinant PHD2 with α-KG at the indicated concentrations. (**C**) Protein expression of HIF-1α and VEGFA in HepG2 cells treated with SA and α-KG. (**D**) HIF-1α

expression in HepG2 cells treated with SA, DMOG and $CoCl_2$. (**E**) The concentrations of α-KG in parental and *GSTZ1*-KO HepG2 cells. Data are shown as mean ± SEM (n = 6 in each group). (**F**) PHD2 expression in *GSTZ1*-KO HepG2 cells treated with or without NTBC. (**G-H**) Co-IP assays to detect the direct interaction between PHD2 and HIF-1α treated with SA in HepG2 and Huh7 cells. (**I**) Co-IP assays to detect the direct interaction between PHD2 and HIF-1α in HepG2 *GSTZ1*-KO and Huh7 *GSTZ1*-OE cells. (**J**) In vitro prolyl hydroxylation of the purified HIF-1α-ODD protein at 0 min and 15 min using lysates from HepG2 cells incubated with SA or DMOG. **(K-M)** DEN/CCl_4-treated mice with or without NTBC administration (8 mg/L in drinking water). Gross appearance of liver tumors (K); The black circle represents nodules of the tumors; Tumor numbers (L) and tumor liver to body weight ratio (M) (n = 5 mice per group). Values represent the mean ± SEM. Statistical analysis was performed using 2-tailed unpaired Student's t test or one-way ANOVA with Tukey's test (L and M), $^{**}p < 0.01$. Abbreviations: SA, succinylacetone; NTBC, 2-(2-nitro-4-trifluoromethylbenzoyl)-1,3 cyclohexanedione; ns, no significant difference.

Supplemental Figure 7

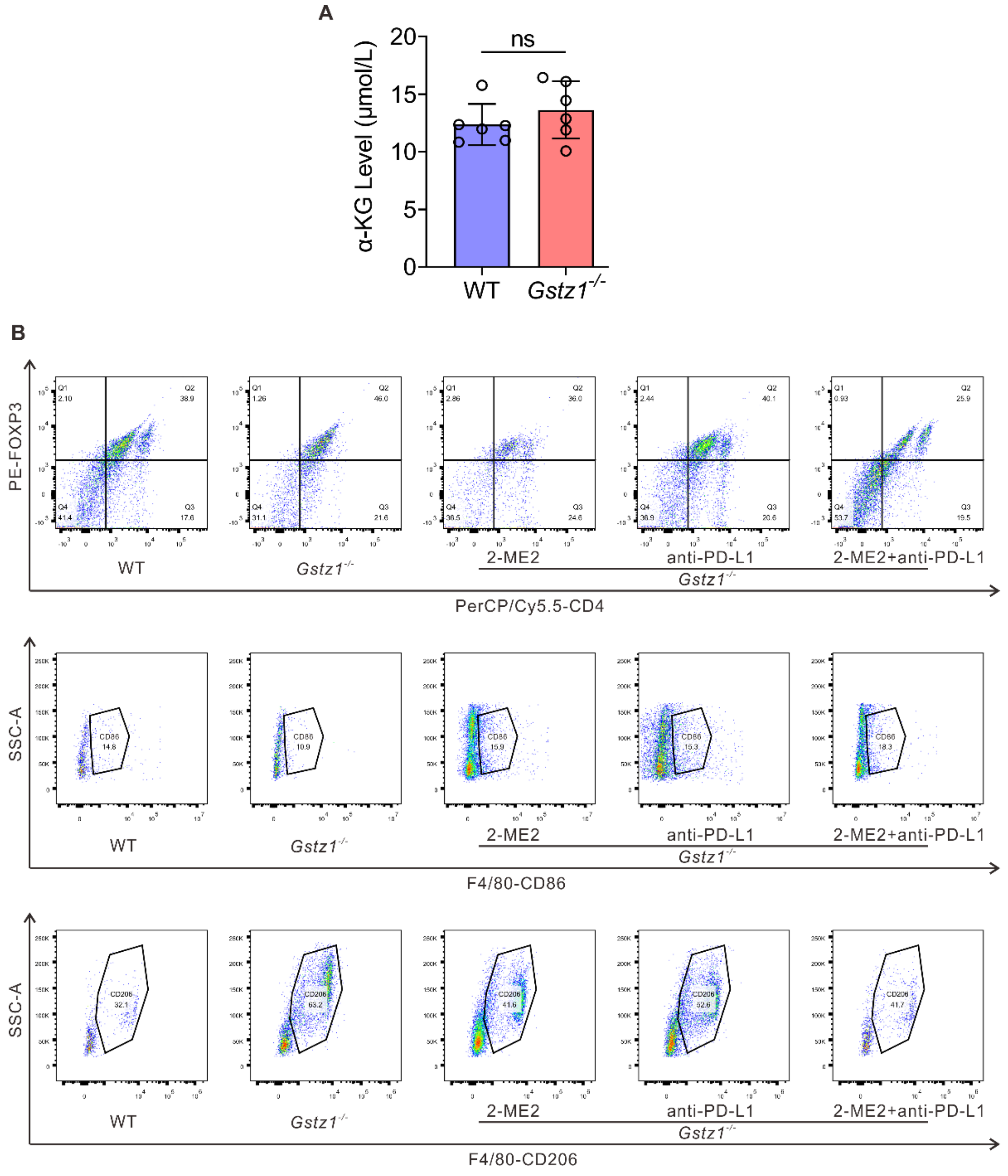

Supplemental Figure 7. Targeting HIF-1α or anti-PD-L1 therapy in *Gstz1*$^{-/-}$ mouse can improve immune effector cell function. (**A**) The concentrations of α-KG in WT and *Gstz1*$^{-/-}$ mouse HCC tissues (n = 6 mice per group). (**B**) The FOXP3^{+} Tregs and M1/M2-like TAMs in the liver tumor of mice were detected by flow cytometry. Statistical analysis was performed using 2-tailed unpaired Student's t test. Abbreviations: NTBC, 2-(2-nitro-4-

trifluoromethylbenzoyl)-1,3-cyclohexanedione; ns, no significant difference.

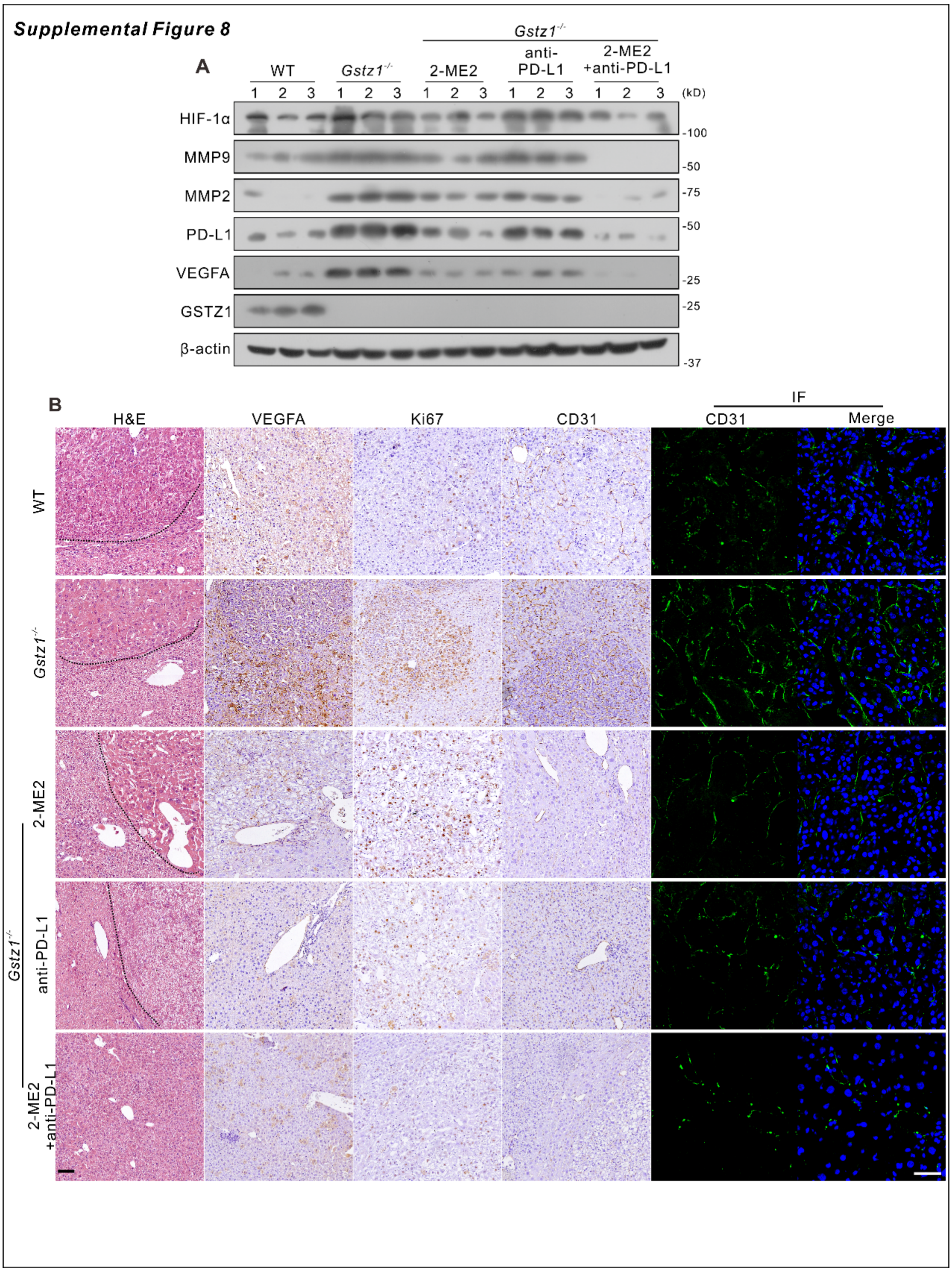

Supplemental Figure 8. HIF-1α inhibitor decreases the expression of downstream target gene. (**A**) The protein expression of HIF-1α, MMP2, MMP9, PD-L1, VEGFA and GSTZ1in three groups of liver tumors were detected by western blot. (**B**) The representative H&E staining (100×), immunohistochemistry (scale bar = 50 μm) and IF (scale bar = 100 μm) images of VEGFA, Ki67 and CD31 in hepatic tumors. Abbreviations: WT, wild-

type; 2-ME2, 2-methoxyestradiol; H&E, hematoxylin and eosin; IF, immunofluorescence.

Full unedited gels for Fig.1F

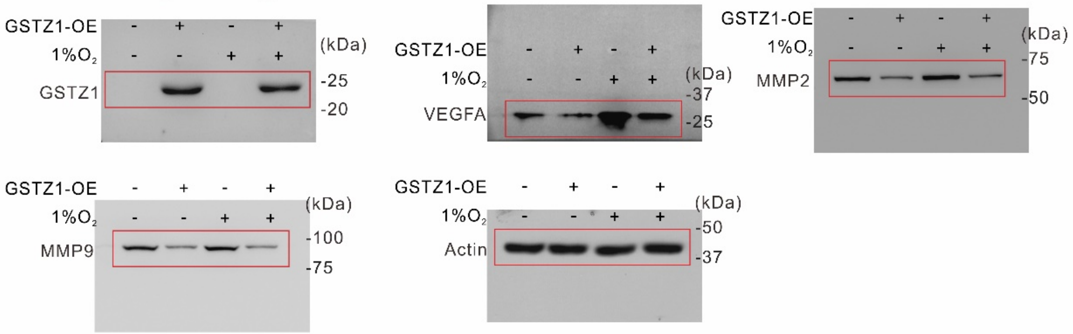

Full unedited gels for Fig.1G

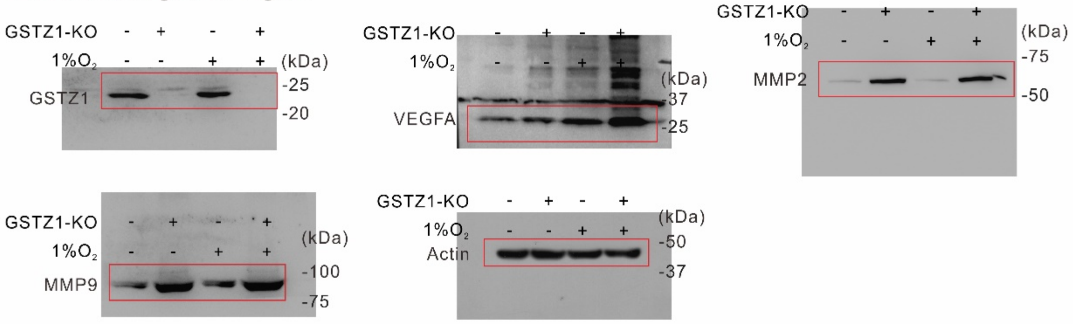

Full unedited gels for Fig.1J

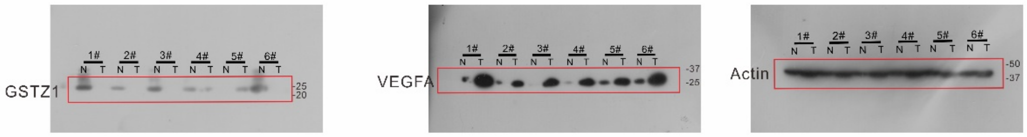

Full unedited gels for Fig.3B

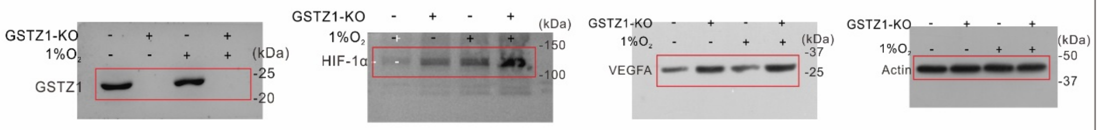

Full unedited gels for Fig.3C

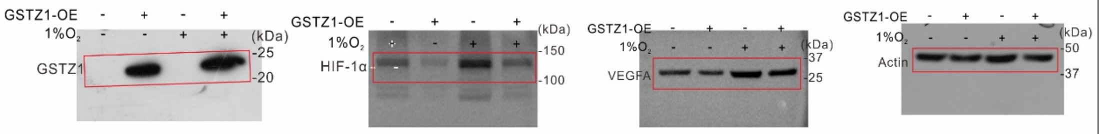

Full unedited gels for Fig.3D

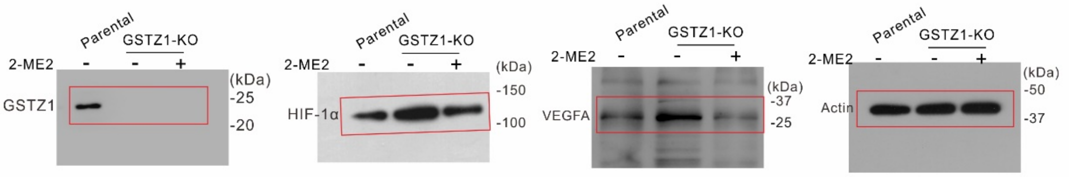

Full unedited gels for Fig.3H

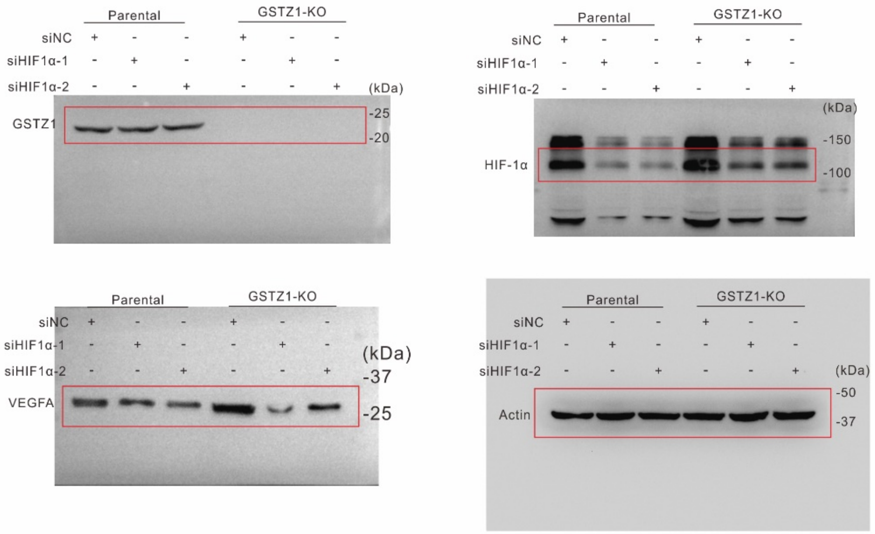

Full unedited gels for Fig.4E

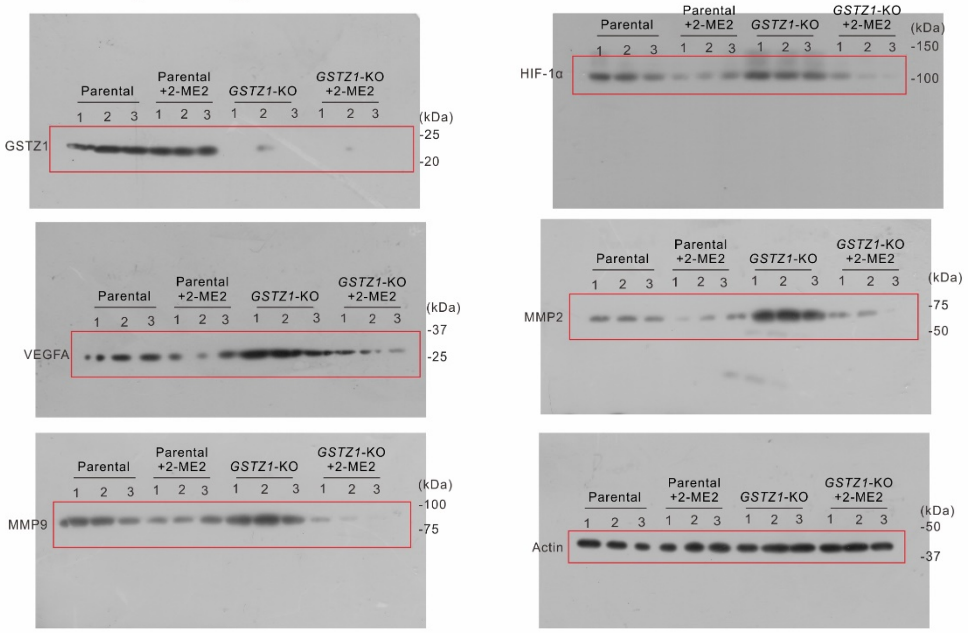

Full unedited gels for Fig.5B

HepG2:

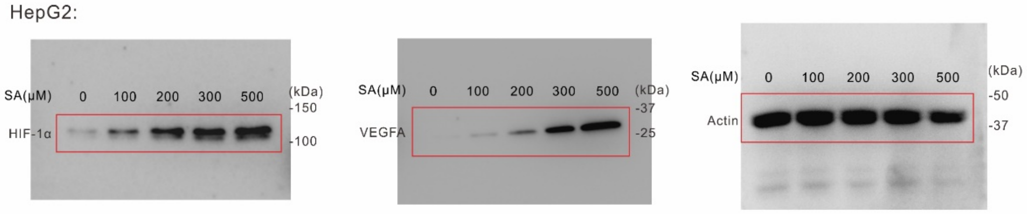

Huh7:

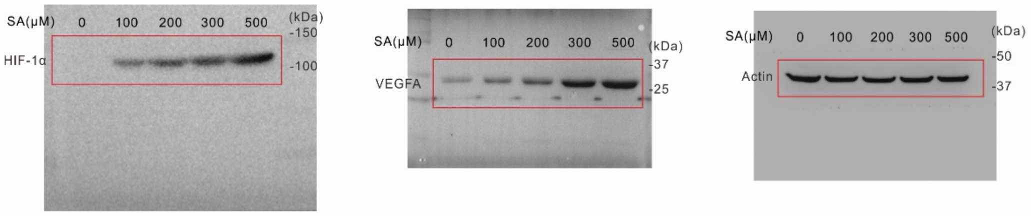

Full unedited gels for Fig.5C

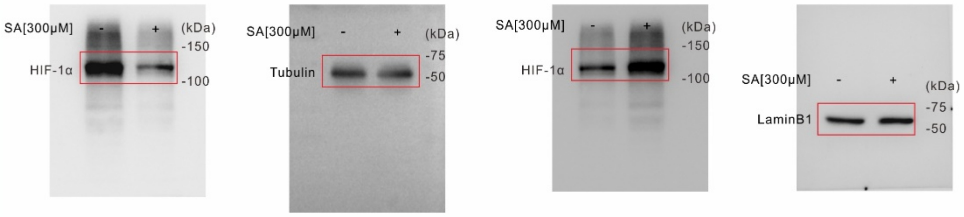

Full unedited gels for Fig.5D

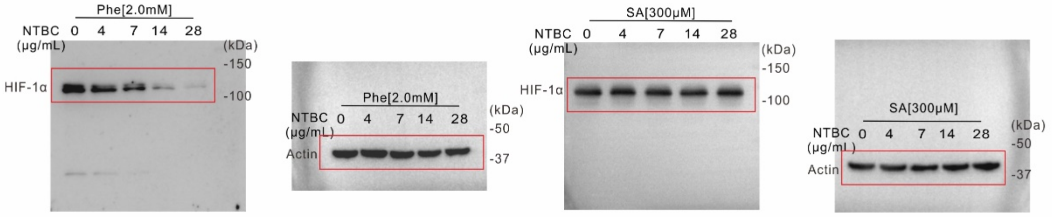

Full unedited gels for Fig.5E

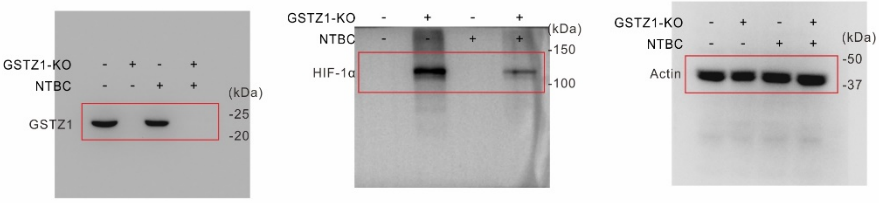

Full unedited gels for Fig.5F

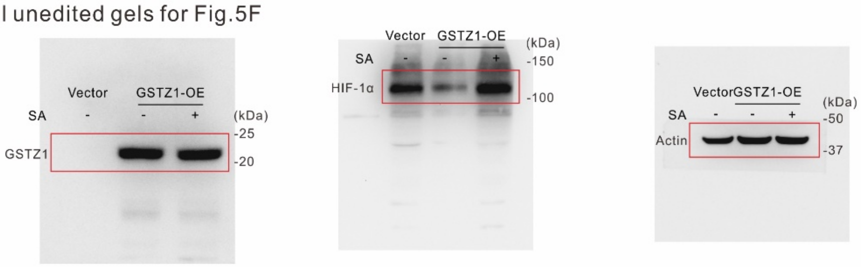

Full unedited gels for Fig.6C

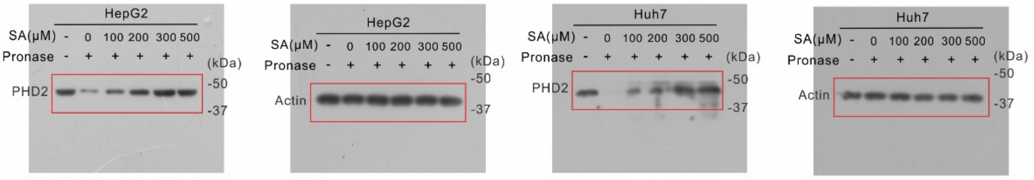

Full unedited gels for Fig.6I

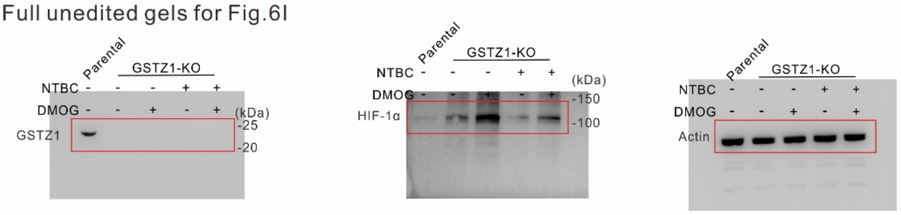

Full unedited gels for Fig.6J

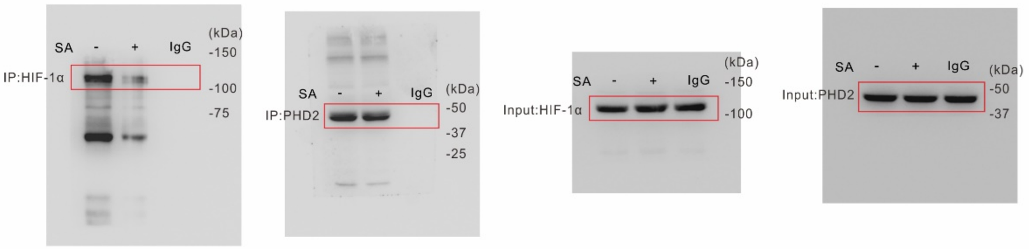

Full unedited gels for Fig.6K

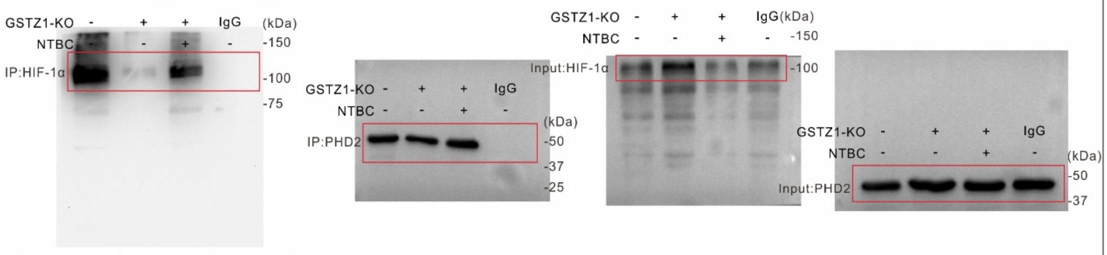

Full unedited gels for Fig.6L

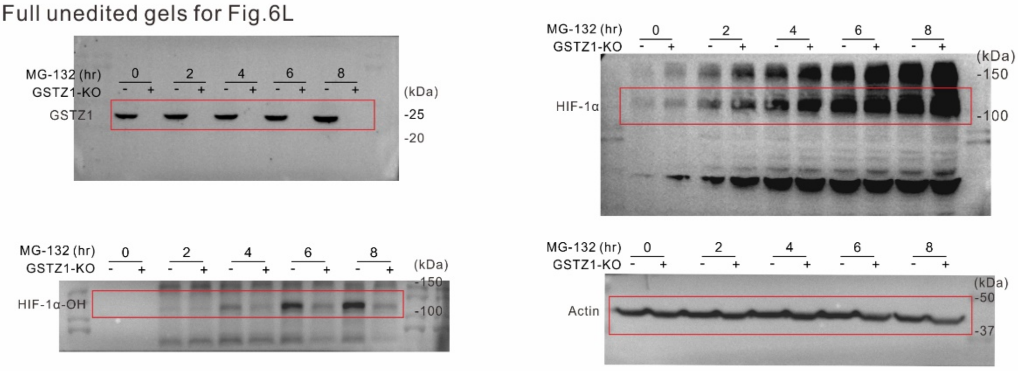

Full unedited gels for Fig.6M

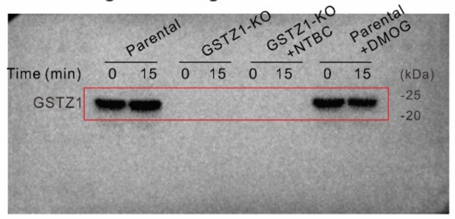

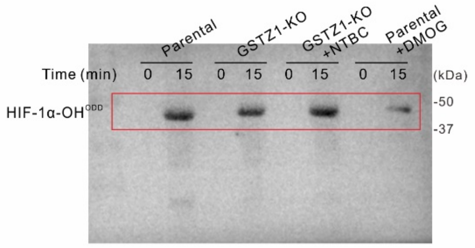

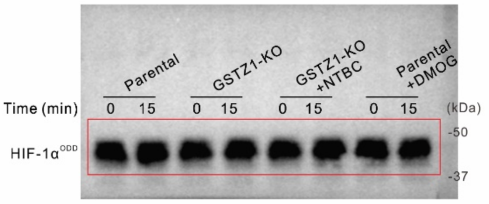

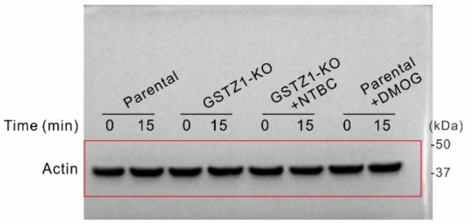

Full unedited gels for S-Fig.1B

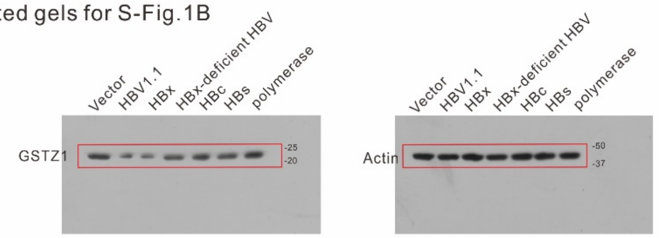

Full unedited gels for S-Fig.1D

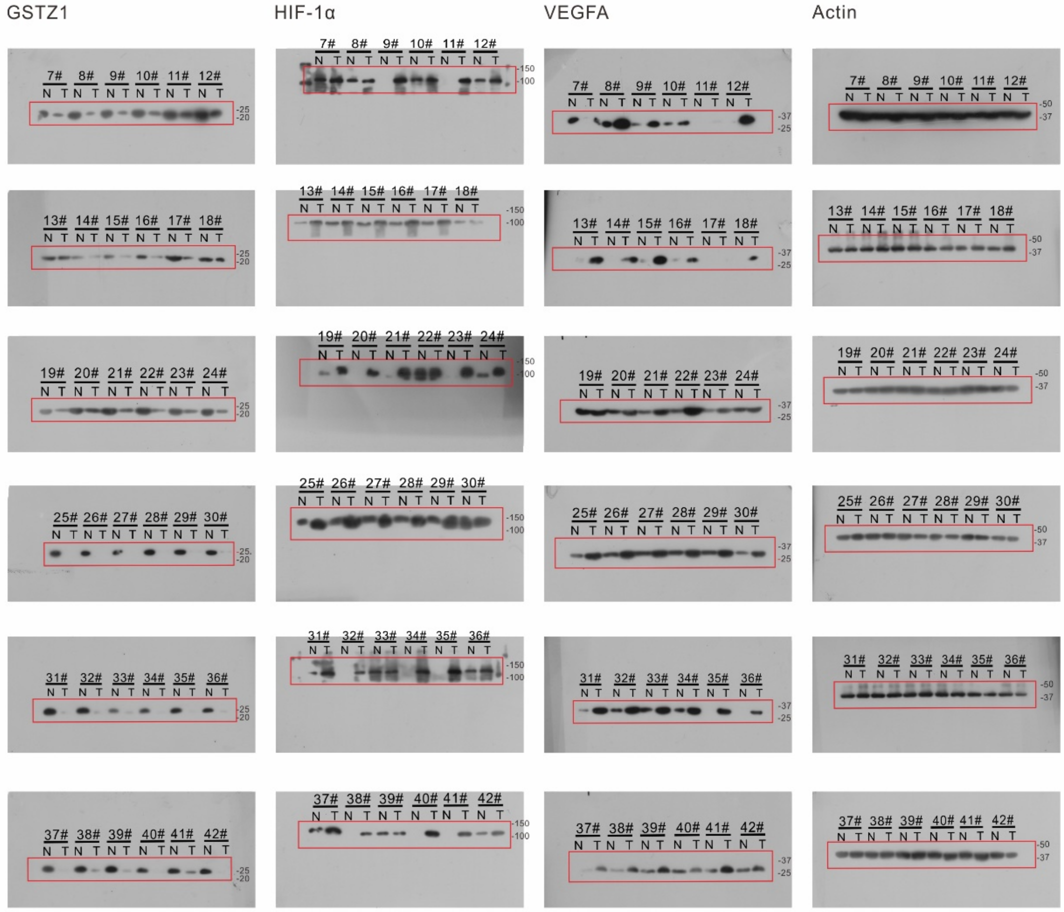

Full unedited gels for S-Fig.3D

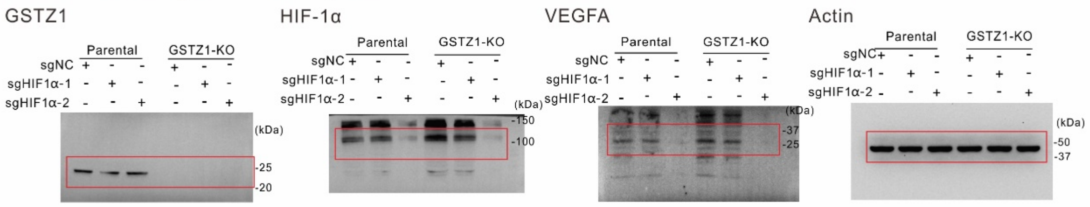

Full unedited gels for S-Fig.5A

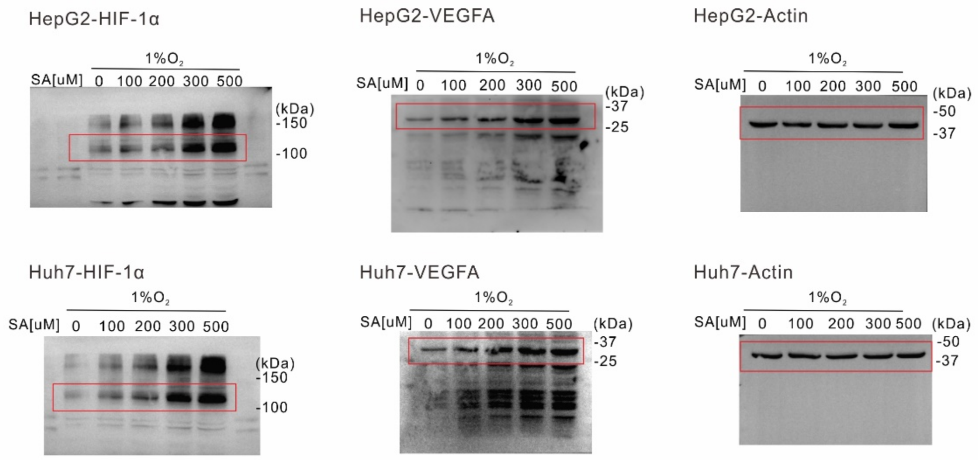

Full unedited gels for S-Fig.6A

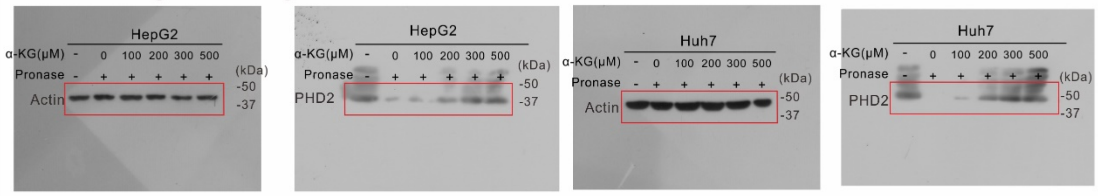

Full unedited gels for S-Fig.6C

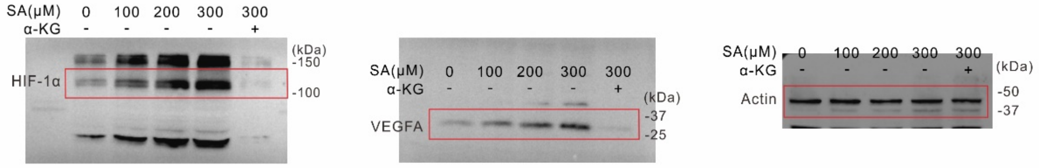

Full unedited gels for S-Fig.6D

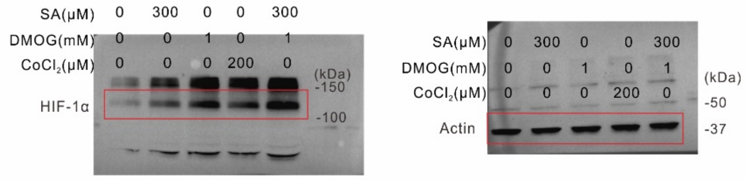

Full unedited gels for S-Fig.6F

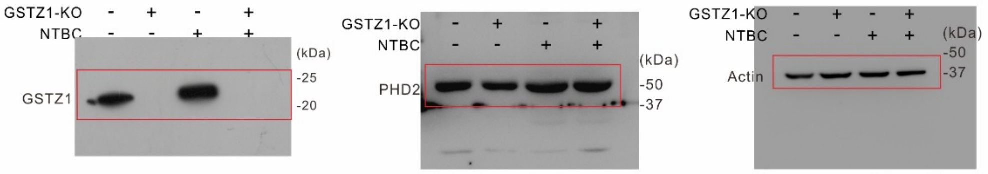

Full unedited gels for S-Fig.6G

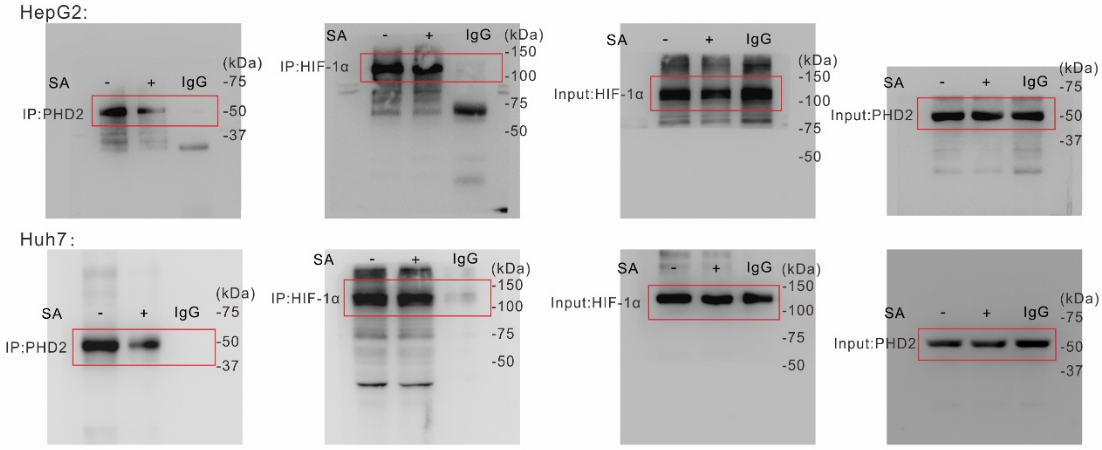

Full unedited gels for S-Fig.6H

Huh7:

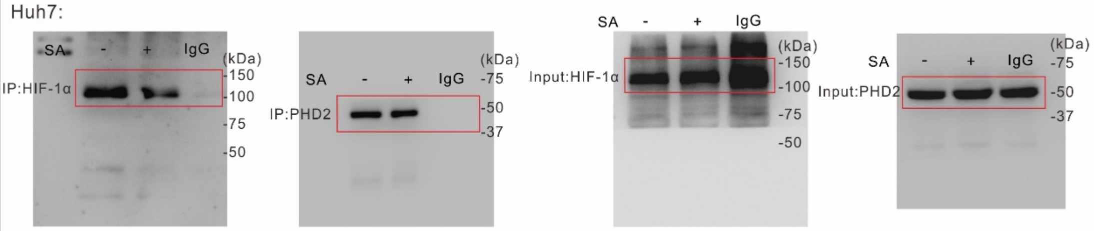

Full unedited gels for S-Fig.6I

HepG2:

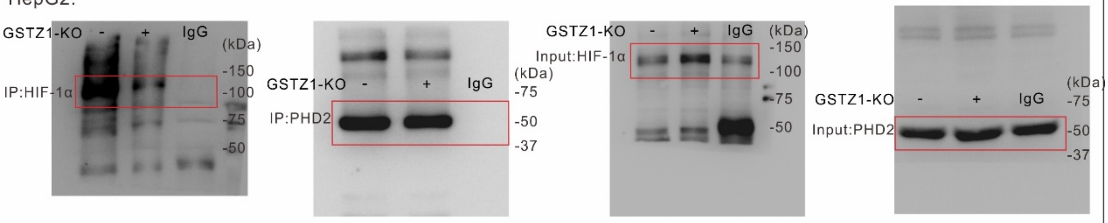

Huh7:

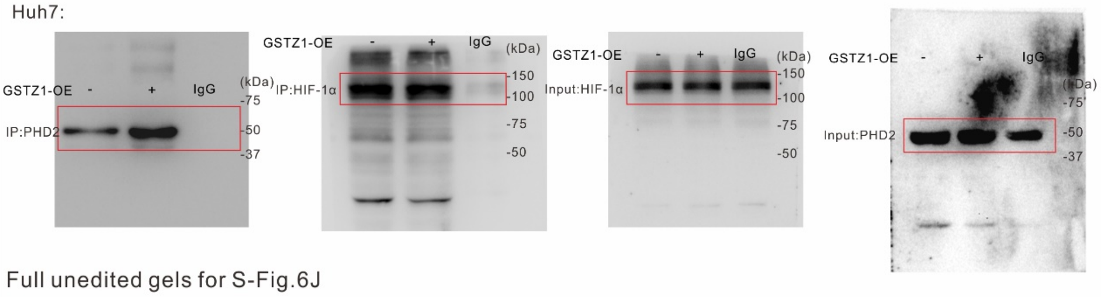

Full unedited gels for S-Fig.6J

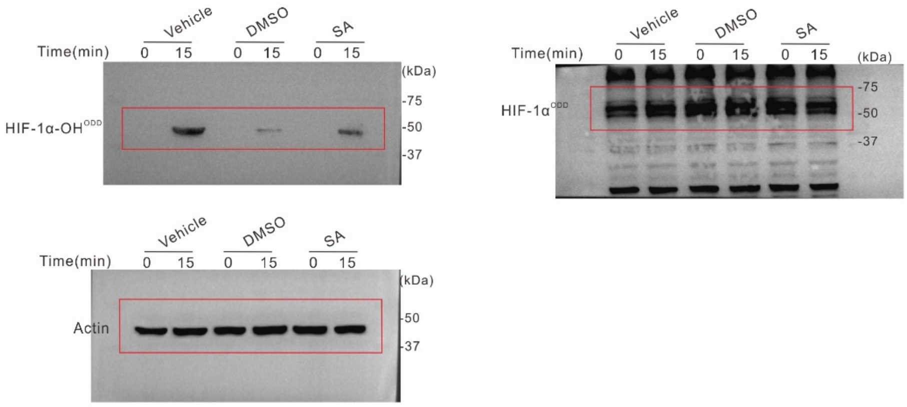

Full unedited gels for S-Fig.8A

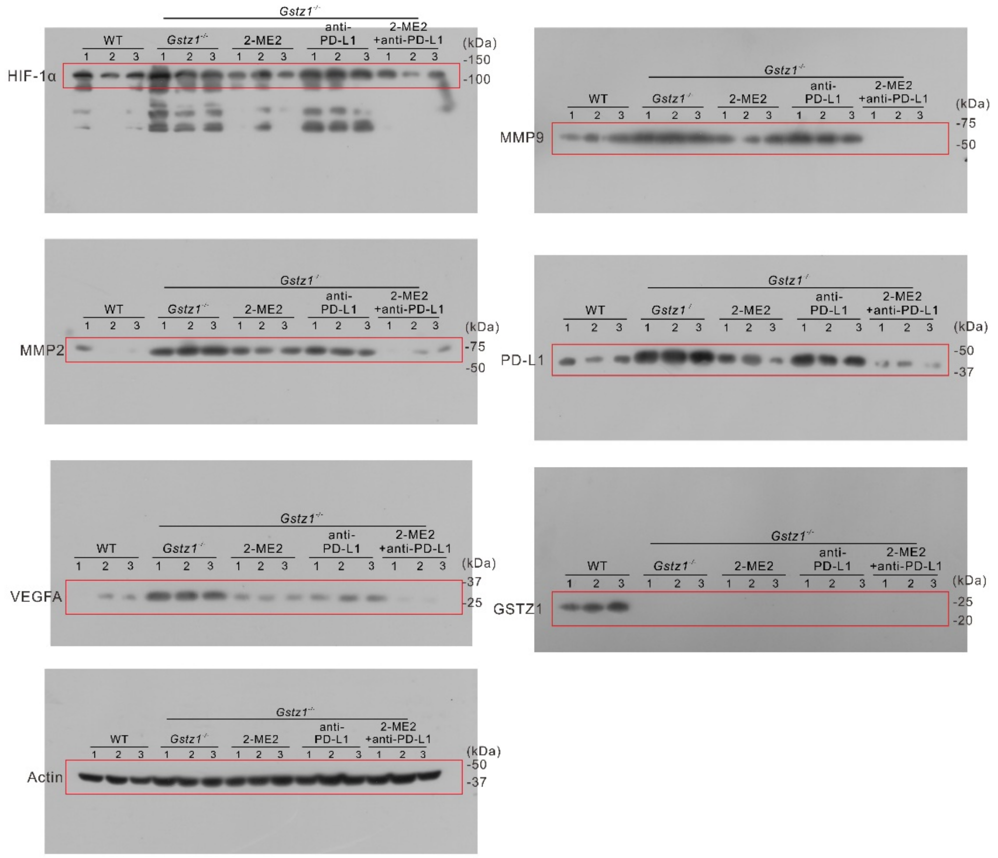

Supplement: Supplemental data [file jciinsight-8-164968-s033.pdf]
